# Reasons for endorsing or rejecting self-binding directives in bipolar disorder: a qualitative study of survey responses from UK service users

**DOI:** 10.1016/S2215-0366(21)00115-2

**Published:** 2021-07

**Authors:** Tania Gergel, Preety Das, Gareth Owen, Lucy Stephenson, Larry Rifkin, Guy Hindley, John Dawson, Alex Ruck Keene

**Affiliations:** a Institute of Psychiatry, Psychology and Neuroscience, King's College London, London, UK; b South London and Maudsley NHS Foundation Trust, London, UK; c NORMENT Norwegian Centre for Mental Disorders Research, University of Oslo, Oslo, Norway; d Faculty of Law, University of Otago, Dunedin, New Zealand

## Abstract

**Background:**

Self-binding directives instruct clinicians to overrule treatment refusal during future severe episodes of illness. These directives are promoted as having the potential to increase autonomy for individuals with severe episodic mental illness. Although lived experience is central to their creation, the views of service users on self-binding directives have not been investigated substantially. This study aimed to explore whether reasons for endorsement, ambivalence, or rejection given by service users with bipolar disorder can address concerns regarding self-binding directives, decision-making capacity, and human rights.

**Methods:**

This qualitative study used data from an internet-based survey distributed to the mailing list of the UK charity Bipolar UK, which contained multiple closed and open questions on advance decision making for patients with bipolar disorder. We included participants who reported that they have been diagnosed with bipolar disorder by a professional (doctor or psychiatrist). In a previous study, quantitative analysis of a closed question about self-binding directives had shown endorsement among a high proportion of participants with bipolar disorder who completed the survey. In this study, we did a thematic analysis of responses from those participants who answered a subsequent open question about reasons for their view. Research was done within a multidisciplinary team, including team members with clinical, legal, and ethical expertise, and lived experience of bipolar disorder. Ideas and methods associated with all these areas of expertise were used in the thematic analysis to gain insight into the thoughts of individuals with bipolar disorder about self-binding directives and associated issues.

**Findings:**

Between Oct 23, 2017, and Dec 5, 2017, 932 individuals with a self-reported clinical diagnosis of bipolar disorder completed the internet survey, with 565 individuals (154 men, 400 women, 11 transgender or other), predominantly white British, providing free-text answers to the open question. 463 (82%) of the 565 participants endorsed self-binding directives, of whom 411 (89%) describing a determinate shift to distorted thinking and decision making when unwell as their key justification. Responses indicating ambivalence (37 [7%) of the 565 responses) were dominated by logistical concerns about the drafting and implementation of self-binding directives, whereas those who rejected self-binding directives (65 [12%] of the 565 responses) cited logistical concerns, validity of their thinking when unwell, and potential contravention of human rights.

**Interpretation:**

This study is, to our knowledge, the first large study assessing the reasons why mental health service users might endorse or reject the use of self-binding directives. The findings provide empirical support for introducing self-binding directives into mental health services as well as advance decision-making practice and policy, and might help address enduring ethical concerns surrounding possible implementation of the directive while a person retains decision-making capacity. The opinions expressed here in responses given by multiple service users with bipolar disorder challenge a prominent view within international disability rights debates that involuntary treatment and recognition of impaired mental capacity constitute inherent human rights violations.

**Funding:**

The Wellcome Trust.

## Introduction

So-called self-binding directives, sometimes known as Ulysses contracts, have been promoted since the 1970s as a means of damage limitation for people with severe episodic mental illness. These directives, which are based on lived experience of harms occurring when treatment is accessed too late, instruct clinicians to overrule treatment refusal during future severe episodes by using involuntary treatment. Following extensive legal and ethical discussion, mental health laws have started to provide for self-binding directives in some jurisdictions in Europe, North America, and Australasia. However, enduring concerns have hindered construction of a widely accepted self-binding directive model that is clinically and legally practicable.^1^, ^2^, ^3^ The main concern is how to ensure that an individual's decision-making capacity for treatment is definitely impaired when a self-binding directive is implemented as a form of early intervention. Although lived experience is central to creating a self-binding directive, evidence regarding service user views about their use is scarce.^4^, ^5^

**Research in context**

**Evidence before this study**

We searched PubMed and Scopus from database inception to Feb 4, 2020, with the broad terms (“self-binding directive” OR “Ulysses” OR “self-binding”) AND (“psychiatric” OR “mental disorder” OR “mental illness” OR “bipolar” OR “mental health”), without any language restrictions. We supplemented the search by reviewing reference lists and forward citations, until no additional relevant articles could be identified. We repeated our searches on Nov 2, 2020, to check for new publications. With the exception of three very small interview-based surveys, all of which included clinicians and service users, research found on self-binding directives as a form of mental health advance decision making was restricted to medico-ethical and legal analysis, despite calls from researchers for empirical work. We found no large studies investigating the views of service users on self-binding directives. There was a paucity of research investigating the views of service users with bipolar disorder on advance decision making in general.

**Added value of this study**

This is, to our knowledge, the first large study to provide empirical evidence about the views of mental health service users on self-binding directives. We found that the majority of service user participants endorsed self-binding directives on the basis that the participants viewed severe episodes of illness as involving a determinate shift and impairment of their thinking and decision making. At the same time, the study includes respondents who rejected this view, demonstrating diversity of opinions among service users with bipolar disorder. This study counters key ethical concerns that have so far prevented the introduction of workable self-binding directives into psychiatric practice and mental health legislation. It also contributes to research on advance decision making in people with bipolar disorder, by supporting the need to consider diverse outcomes and to incorporate empirical studies of stakeholder views.

**Implications of all the available evidence**

The endorsement by the majority of service user respondents of involuntary treatment on the basis of impaired decision-making abilities counters a widespread view, upheld by the UN Committee on the Rights of Persons with Disabilities, that psychiatric use of capacity assessment and involuntary treatment necessarily violate fundamental human rights. Researchers, clinicians, and policy makers should consider that some service users with severe mental health conditions wish to request their own future involuntary treatment, using self-binding directives as a way to self-manage their illness and increase autonomy. When assessing the ethical viability of self-binding directives, mental capacity, and involuntary treatment, human rights advocates need to take a broad range of service user views into account.

Service users drafting self-binding directives usually accept that: severe illness impairs decision-making capacity for treatment; future treatment refusal might cause them harm; and involuntary treatment potentially offers protection, control, and increased autonomy.^1^ Although a small body of research examining service users' retrospective views of involuntary treatment found that a substantial proportion thought it was justified,^6^, ^7^, ^8^ involuntary treatment and substitute decision making—ie, when decisions relating to a person whose decision-making capacity is judged to be impaired are made by another person—are generally seen as deeply controversial.^9^ In particular, the UN Committee on the Rights of Persons with Disabilities rejects the notion of impaired decision-making capacity and has called for complete abolition of involuntary treatment.^10^

This study aims to understand how these ideas relate to highly influential human rights and ethical assumptions surrounding autonomy and psychiatry. We examine whether reasons given by service users with bipolar disorder for endorsing or rejecting self-binding directives provide empirical support for the use of such directives and address concerns specific to self-binding directives, such as worries about implementation before the loss of decision-making capacity, and broader human rights concerns. We focused on people with bipolar disorder because severe periods of bipolar illness are typically episodic and repetitive and often feature a loss of decision-making capacity for treatment that is regained during recovery, and because there is a paucity of research on advance decision making for this condition.^11^, ^12^

## Methods

### Study design and participants

For the original survey, our target population comprised 20 134 people on the mailing list of Bipolar UK, the UK's largest charity dedicated to bipolar disorder. These 20 134 people had registered their email and provided consent to be contacted by the charity at the time of distribution. On Oct 23, 2017, Bipolar UK sent a dedicated email containing the URL to the online questionnaire with a description of the project and a request for participants. The survey remained open until Dec 5, 2017. Bipolar UK continued to promote the survey via social media, monthly newsletters, a reminder email, and direct communication via support groups throughout the 6-week period to maximise response rate. Respondents were able to revisit pages already completed and edit responses, and only completed questionnaires could be accessed for analysis by the research team. The internet survey medium was helpful for ensuring wide distribution and facilitating privacy and full anonymity when answering questions concerning such sensitive subject matter. Inclusion criteria were having provided informed consent and being either a person with, or a carer for a person with, a self-reported diagnosis of bipolar disorder by an appropriate professional (doctor or psychologist).

Informed consent was sought from potential participants before the start of the survey. Participants were given the opportunity to provide personal email addresses if they wanted to receive more information about the project in the future. These participants were uncoupled from the data before the analysis to prevent loss of anonymity. No other identifying information was sought in the questionnaire. Ethics approval was provided by the London–Surrey Borders Research Ethics Committee and Health Research Authority (REC reference number 17/LO/1071).

### Procedures

The exploratory survey asked about experiences and attitudes towards advance decision making. The survey included closed and open (free-text) questions. A brief introductory section included a simple explanation of advance care planning and existing provision within the Mental Capacity Act 2005 (England and Wales) for formal advance decision making. No other background information was included. The questionnaire can be accessed online. The quantitative analysis of all answers from the service users to closed questions and a detailed description of the survey's rationale and methods have been published previously.^11^

The survey was designed following a review of the literature. First, a pilot questionnaire was formulated and reviewed by the research team. The revised questionnaire was then reviewed by two experienced Bipolar UK employees before being piloted on eight people with bipolar disorder and five carers who provided written feedback. Follow-up interviews were done with one of the service users and one of the carers to discuss the feedback in detail and trial possible alterations. The questionnaire was revised a second time by the original research team (GH, TG, LS, GO, LR, ARK). Both the original survey research team and extended research team (all authors) for the current study included individuals with expertise in psychiatry, medical ethics, law, psychotherapy, and lived experience of advance decision making for people with bipolar disorder, allowing multidisciplinary and coproduced study design and analysis throughout.

One closed question was: “Some people think a ‘self-binding statement’ is a good idea. This states that the person wants the contents of their advance care plan to be respected even if they no longer agree with it during an episode of illness. Do you think this is a good idea?”. The wording “Some people think a ‘self-binding statement’ is a good idea” was chosen as a means to stimulate critical thinking and is indicative of the positive general consensus among available discussions about the idea of self-binding directives, despite various concerns. As a response, participants filled out a Likert-type scale from 1 to 5 with the following options: (1) “definitely yes”, (2) “probably yes”, (3) “neither yes nor no”, (4) “probably no”, and (5) “definitely no”. For the current study, we classified closed answers as: (1) and (2) indicating endorsement (hereafter referred to as the endorsement group), (3) indicating ambivalence (hereafter referred to as the ambivalence group), and (4) and (5) indicating rejection (hereafter referred to as the rejection group). The results showed high levels of endorsement, with 719 (77%) of 932 participants expressing endorsement, 120 (13%) expressing ambivalence, and 90 (10%) expressing rejection, and only three participants did not answer.^11^

A further open question, “Why do you think this is?”, invited free-text responses, without a fixed word limit. The aim of the current study was to understand more about reasons for endorsement, rejection, or ambivalence, by analysing the free-text answers.

### Data analysis

Given the complexity of the self-binding directive concept, we excluded free-text responses in which factors such as brevity or apparent incongruity between quantitative and qualitative responses made it unclear whether the concept had been understood. Qualitative free-text answers were assessed both independently and then in terms of congruity with the classification of the quantitative answers. PD and TG did the initial independent checks of all answers and their initial analysis aligned almost completely. PD and TG then discussed together and reached agreement concerning the small number of cases that remained uncertain (56 of 621 answers), and the classification of all answers was circulated among LS, GO, ARK, and LR, to check until final agreement was reached.

Although usually short, the free-text answers, linked directly to the quantitative endorsement question, provided a conceptually rich and varied dataset, and we used thematic analysis to identify themes within it.^13^ To emphasise distinct patterns emerging from the data, we combined in-depth conceptual analysis of inductively derived themes with quantitative analysis of theme distribution, following established methods for analysing open survey responses.^14^, ^15^

Data were entered into coding software (NVivo 12). PD and TG read the raw data independently, discussed initial reflections, and then developed a preliminary coding framework. An inductive approach was used and both the coding framework and the themes were refined through an iterative process, including regular consultation with all authors, until saturation was reached. The data for each theme were then checked several times by both PD and TG to ensure conceptual coherence, and final percentages for distribution of each theme within the endorsement, ambivalence, and rejection groups were calculated.

### Role of the funding source

The funder of the study had no role in study design, data collection, data analysis, data interpretation, or writing of the report.

## Results

Between Oct 23, 2017, and Dec 5, 2017, the questionnaire was sent to 20 134 people and accessed by 3418 people, with 1131 people completing the questionnaire or a parallel carer's questionnaire, constituting a response rate of 5·61%. This rate was the combined response rate for both service users and carers, given that the total number of service users and carers among the target population was unknown. 50 completed service user questionnaires were excluded because of absence of consent or not meeting the diagnostic inclusion criteria. This exclusion left a study population of 932 service users with bipolar disorder, of whom 621 answered the free-text question about self-binding directives. After data quality checks, 56 (9%) of the 621 responses were excluded, leaving a dataset of 565 service user responses, divided into endorsement, ambivalence, and rejection groups. Response rates for the free-text question were high among participants who endorsed (463 [64%] of 719 responders) and rejected (65 [72%] of 90 responders), but lower among those who were ambivalent (37 [31%] of 120 responders).

565 (61%) of the 932 participants provided reasons behind their answers to the closed question about endorsement of the self-binding directives concept. These participants had self-identified as having a diagnosis of bipolar disorder and were predominantly white British, women, and well educated (table 1). Many respondents had lived experience of severe illness and treatment for which self-binding directives would apply. A majority (352 [62%]) reported at least one episode of hospitalisation, a third (193 [34%]) reported at least one experience of formal involuntary detention, and many mentioned experiences of mania, psychosis, and suicidality. The proportion of respondents reporting hospitalisation experiences within the endorsement, ambivalence, and rejection groups was similar, with slightly higher rates of previous detention among those who endorsed or were ambivalent than among those who rejected the idea of self-binding statements. The distribution of hospitalisation rates was 291 (63%) of 463 respondents in the endorsement group, 24 (65%) of 37 respondents in the ambivalence group, and 37 (57%) of 65 respondents in the rejection group. For detention rates it was 165 (36%) of 463 respondents in the endorsement group, 12 (32%) of 37 respondents in the ambivalence group, and 16 (25%) of 65 respondents in the rejection group.

**Table 1:** Demographical details of the survey population

	**Survey population (n=565)**
**Age, years**	
Mean (SD)	47·7 (12·5)
No response	30
**Gender**	
Men	154
Women	400
Transgender	2
Other	2
Prefer not to say	4
No response	3
**Ethnicity**	
Arab	1
Bangladeshi	0
Black African	0
Black British	3
Black Caribbean	3
Chinese	1
Gypsy or Romany	0
Indian	2
Latin American	1
Mixed White and Asian	3
Mixed White and Black African	1
Mixed White and Black Caribbean	2
Other mixed	10
Other Asian	1
Other Black	0
Other White	33
Other	5
Pakistani	1
White British	492
No response	6
**Relationship status**	
Single, not in a relationship	127
Married or civil partnership	237
In a relationship and living with partner	62
In a relationship, not living with partner	51
Separated	17
Divorced	60
Widowed	7
Other	0
No response	4
**Highest education**	
No formal qualification	19
GCSE or equivalent	63
A Level or equivalent	70
Undergraduate degree or diploma	208
Postgraduate qualification	173
Other	28
No response	4
**Employment**	
Full time	121
Part time	93
Casual work	13
Student	22
Unemployed	35
Long-term sickness or disability	147
Retired	81
Carer	4
Looking after family or home	22
Other	22
No response	5
**English as first language**	
Yes	541
No	17
No response	7
**Country of residence**	
England	478
Scotland	19
Wales	43
Northern Ireland	7
Other	12
No response	6
**Region of UK**	
Southwest	76
Southeast	99
London	78
East of England	40
East Midlands	27
West Midlands	40
Yorkshire and the Humber	36
Northwest	53
Northeast	31
No response	85
**Number of hospitalisations due to mental health**	
0	206
1–2	170
3–4	91
5 or more	91
Do not know	5
No response	2
**Detention under the Mental Health Act**	
0	365
1–2	125
3–4	48
5 or more	20
No response	7
**Comorbidity**	
Yes	310
No	248
No response	7
**Comorbidity type**	
Alcohol or substance misuse	49
Anxiety	258
Depression	243
Panic disorder	56
Obsessive compulsive disorder	45
Post-traumatic stress disorder	62
Schizophrenia	8
Schizoaffective disorder	24
Eating disorder	40
Attention-deficit hyperactivity disorder	12
Emotionally unstable personality disorder	49
Other personality disorder	24
Other	15
**Time since diagnosis, years**	
Mean (SD)	12·9 (11·1)
No response	7

Data are n or mean (SD). GCSE=General Certificate of Secondary Education (in the UK except Scotland).

Although 463 (82%) of the 565 participants endorsed self-binding directives, support was not unanimous, with 37 (7%) participants being ambivalent and 65 (12%) rejecting (table 2). Five clear themes concerning reasons for endorsement, ambivalence, or rejection emerged. Reasons for endorsing self-binding directives were classified into two themes: distorted thinking when unwell and benefits. Reasons for rejecting or questioning self-binding directives were classified into three themes: logistical concerns relating to drafting and implementation, valid thinking when unwell, and harms. Both the benefits and harms themes were subdivided into conceptual and practical subthemes, to differentiate between outcomes such as increased or decreased empowerment (conceptual) and reduced suicide risk (practical).

**Table 2:** Distribution of themes and subthemes within response groups

	**Endorsement group (n=463)**	**Ambivalence group (n=37)**	**Rejection group (n=65)**
**Thinking when unwell**			
Distorted thinking when unwell	411 (89%)	8 (22%)	8 (12%)
Valid thinking when unwell	7 (2%)	5 (14%)	26 (40%)
**Benefits**			
Subtheme: practical benefits	58 (13%)	2 (5%)	1 (2%)
Subtheme: conceptual benefits	36 (8%)	1 (3%)	0
**Harms and concerns**			
Subtheme: practical harms	7 (2%)	8 (22%)	6 (9%)
Subtheme: conceptual harms	1 (<1%)	7 (19%)	26 (40%)
Logistical concerns	63 (14%)	36 (97%)	33 (51%)

Given that many individual responses contained multiple themes, the total percentages for distribution of themes within each group exceed 100%.

The most striking result was the predominance of the distorted thinking when unwell theme (table 2) and the remarkably varied articulations of this idea (panel; see appendix pp 1–23 for full dataset).

Panel **Quotations supporting themes and subthemes**

**Distorted thinking when unwell**

• “Being well and not being well are quite different states. Being not well puts one in a tricky place for making good decisions.” (endorsement group)
• “I feel an individual's wishes should be respected although there is a point when an individual becomes too unwell and cannot make decisions for themselves.” (rejection group)
• “If it has been planned during well periods then that is a true representation of wishes.” (endorsement group)
• “You are unwell and lack capacity. I would agree with this statement looking back at when I was last sectioned but my views were very different at the time due to my illness. It is my well views and opinions that should be acted upon.” (endorsement group)
• “I wouldn't have the capacity to make careful, reasoned judgements when unwell.” (endorsement group)
• “When I am ill, I am not of the right mind. So making a decision at this time would probably not have a good outcome.” (endorsement group) or “I think this because when I'm unwell I would not be making the right decisions for myself and my wellbeing.” (endorsement group)
• “I think that it should be respected as when I'm unwell I make unwise decisions.” (endorsement group)
• “Whilst unwell it is very common to have ideas that are not in one's best interest.” (endorsement group)
• “Because when I'm in a depressive state I want to die, when I'm not I don't. I'm a different person with different thoughts, feelings and reactions when I'm depressed or even manic, it wouldn't be fair if I'd stated in my care plan to persist in treatment to get better but was refused because of my depressed or manic state.” (endorsement group)
• “Because psychosis intrinsically, temporarily, alters one's judgement.” (endorsement group)
• “Because losing insight into one's illness and experiencing resistance to treatment are such key features of bipolar episodes for so many people, and lack of treatment at these points can lead to harmful behaviours if treatment is not imposed.” (endorsement group)
• “Because when I am unwell I am irrational and unable to make healthy or safe decisions.” (endorsement group)
• “During episodes you lose the objectivity that is present when well. It helps if such objectivity is self-binding during periods of illness, provided the circumstances have been foreseen.” (endorsement group)
• “When psychotic or manic or depressed you can become another person and irrational. It is easy to make bad decisions when ill, that may not be in my best interest. You cannot have the lunatics running the asylum? Can we?” (endorsement group)
• “When we have an episode of high or low mood, we are not ourselves. We do not think straight.” (endorsement group)
• “When I am ill, all I see is the pain and torment of my existence, I want to die and any easy way would be seized upon and refusal of medication which I would do just cements these ideas. If I make a care plan whilst feeling normal I know I am far happier, contented and much more rational and l know that it would be bad for me and my family if I died. This is why I believe a care plan would work. At least then there is something in writing that I know is a true reflection of my life rather than the warped one my brain insists is true.” (endorsement group)

**Conceptual benefits**

• “Gives people voices before they are ill and unable to make them.” (endorsement group)
• “If it has been planned during well periods then that is a true representation of wishes.” (endorsement group)

**Practical benefits**

• “When feeling and thinking clearly, you can agree to sensible ideas of what type of care you may need. However, during an episode—manic or depressive—you don't think the same and although you agreed certain things when well, it may be very difficult to deal with these decisions as they could be traumatic when unwell, even if they are there to help you get better.” (endorsement group)
• “Because when I am unwell, my thoughts, perceptions and desires drastically change. I can become suicidal and impulsive, making decisions or plans that I (would) later regret (if I had followed through with them). I often come out of an episode feeling extraordinarily grateful that I didn't take my own life, but when I become ill the same thoughts and plans return to me regardless and I am convinced they are my true desires. I don't want anyone to listen to me when I am unwell, I explicitly would want them to take my advance plans as my true wishes and would be mortified if they were overruled.” (endorsement group)

**Logistical concerns**

• “Could have changed between making and becoming so unwell, but not recorded...due to practicalities of life, and thus may not be true wishes.” (ambivalence group)
• “Things change and sometimes it's difficult to guess what might happen in a hypothetical situation.” (ambivalence group)
• “How do you know they were of fit mind when they wrote it? How do you know someone else has not befriended that person for financial gain?” (ambivalence group)
• “It is hard to say. It depends how unwell you are in a particular circumstance. I have been unwell but still perfectly able to make my own decisions and on occasions I have been so unwell I've not been able to make my own decisions.” (ambivalence group)
• “It's so hard with bipolar and my family are very controlling. I am afraid what could happen and knowing I were committed to it forever could be daunting.” (rejection group)

**Valid thinking when unwell**

• “I am wary of being hospitalised when I am manic, because I do not feel that this is a destructive part of my illness: in fact, I enjoy it, am happy, productive, and move my life on in important ways, ways which I can't do in hospital. However, I am well aware that psychiatric professionals all see mania as very destructive, even though my most destructive behaviour is by far when I am severely depressed. So, I assume that a psychiatrist would probably attempt to get me to agree to hospitalisation or treatment for manic episodes as well as depressive, which I do not want, but could not convince a psychiatrist of.” (rejection group)
• “Were I manic I might still have insight, were I psychotic probably not.” (ambivalence group)

**Conceptual harms**

• “Not empowering or respectful.” (rejection group)
• “Even when I'm mad, I'm still a human and have the right to make decisions even if they are bad ones.” (rejection group)

**Practical harms**

• “If a person says they wanted to stay home to be treated but are clearly a danger to themselves or others, hospital and care is the best place.” (rejection group)
• “It depends how ill you are. Docs sometimes have to make decisions in difficult circumstances in order to help you. A self-binding statement could get in the way. Then there is the question of legal responsibility and liability.” (ambivalence group)

A key reason for endorsing self-binding directives was the theme of distorted thinking when unwell. This theme appeared in nearly all endorsements (411 [89%] of the 463 endorsements), and even within some rejection and ambivalence responses. The term distorted, used by a few participants, was chosen for this theme's name, to capture the central idea of adverse change in thought processes, without the distinct judgment conveyed by terms such as invalid. This adverse change was presented as a substantial alteration from valid thinking when well and was generally described as a determinate shift that substantially compromised decision-making abilities. A response example from the endorsement group was: “A person going through a manic episode has, by definition, seriously distorted thinking. If a person is manic to the extent that they cannot make their own decisions then again, by definition, they are not in a right mind to refute their advance care plan—otherwise it's basically worthless.”

Thought processes when well were presented as valid or authentic, either explicitly or implicitly. Participants linked this idea to using lived experience of previous episodes of severe illness to understand both the effect of distorted thinking when unwell on decision-making abilities and how to manage future episodes. Many respondents stated explicitly that the breakdown of decision-making abilities justifies overruling their treatment decisions “during an episode of illness”. Some of the free-text answers referred to outcomes of decision making, rather than simply the process, and used language clearly implying value judgments, such as best, right, good, or wise decision. The effect on decision-making ability and outcomes was expressed with great variety, ranging from the appearance of directly evoking medicolegal terminology associated with decision-making capacity to using far less technical language.

Many participants used diagnostic terms like mania, psychosis, and depression when explaining the distorted thinking and shift from valid to distorted thinking when becoming unwell, whereas others used the concept from psychiatry of lack of insight into one's illness. Many responses conveyed distortion through stark descriptions of so-called irrationality, using highly emotive and even stigmatising language. Such responses might represent a deliberately hyperbolic attempt to distance themselves from their so-called ill self—a distancing also expressed by presenting distortion as a fundamental change of identity.

Some respondents presented the benefits (theme) of self-binding directives as a reason for endorsing their use and suggest potential positive outcomes. 36 (8%) of the 463 participants who endorsed the use of such directives mentioned conceptual benefits (subtheme) as a justification, such as increased empowerment, autonomy, authenticity, and rights, and there was substantial overlap with the theme of distorted thinking when unwell. For example, one response from the endorsement group was “I use it myself. I give mental health staff permission to section me if needed. It gives back the power to me.”

58 (13%) of the 463 participants who endorsed the use of self-binding directives suggested that these directives might bring them potential practical benefits (subtheme of benefits), including ensuring treatment, minimising risk, and collaboration in, and continuity of, care. One example response from the endorsement group was: “When you are in either a manic or depressive episode you are more likely to stop people helping and just want to push away any help.” Some respondents described the cost–benefit analysis of weighing the consequences of not receiving treatment against the probable trauma of experiencing involuntary treatment (panel; appendix pp 8, 18). Eight participants explicitly presented self-binding directives as a way to reduce suicide risk. Some free-text answers were quite closely connected to the theme of distorted thinking when unwell.

A small group of participants rejected self-binding directives (65 [12%] of 565 participants) or were ambivalent about their use (37 [7%] of 565 participants), expressing doubts about the viability of such directives and the prospect of potential harms, which often directly conflicted with reasons given for endorsement.

The theme of logistical concerns about the drafting and implementation of self-binding directives was the most prevalent among participants who gave ambivalent (36 [97%] of 37 participants) and rejection responses (33 [51%] of 65 participants), and also appeared in responses of participants who endorsed the use of self-binding directives (63 [14%] of 463 participants). Many participants expressed concerns about the criteria for valid advance decision making, such as being properly informed. A common concern was that the self-binding directive would become out of date, as illustrated in a response in the rejection group: “A plan that is written when I am well is a good idea BUT I may have changed my mind about things and not updated the original plan.” Some respondents worried about the applicability of the self-binding directive to later circumstances, when they are unwell. Seven worried about ensuring decision-making capacity at the time the directive was completed. Six participants from the ambivalence or rejection groups, along with six participants from the endorsement group, voiced concerns about ensuring that decision-making capacity for treatment was definitely impaired at the time of implementation. Four participants expressed concern about undue influence from family, clinicians, or others.

The theme of valid thinking when unwell was given as a reason for rejecting self-binding directives by 26 (40%) of the 65 participants who rejected the use of such directives; the term “valid” was used in several responses and thus was incorporated in the theme name. The idea of valid thinking when unwell seemed to directly challenge the theme of distortion of thinking when unwell. An example from the rejection group was: “I think how a person is feeling in a crisis is valid. As it's valid at the time, the advanced statement might be temporarily invalid.” One powerful statement presented mania as a valid and positive dimension of the participant's life, contrary to the usual psychiatric evaluation. Another concern was that valid thinking might persist through some severe illness states, but not others, whereas the self-binding directive would apply during all. A small proportion (five [14%] of 37 participants) who gave ambivalent responses and even a few of those who endorsed the use of self-binding directives (seven [2%] of 463 participants) shared these valid thinking reservations, without being convinced by them, illustrating the complexities of the cost–benefit analysis.

Some respondents presented the potential harms (theme) of self-binding directives as a reason for rejection or ambivalence regarding the use of self-binding directives, suggesting that the use of such directives could have damaging outcomes. 26 (40%) of the 65 participants who rejected the use and seven (19%) of the 37 participants who were ambivalent raised concerns about conceptual harms (subtheme), presenting a distinct idea: that, although thought processes might change when a person is unwell, this alteration does not necessarily render the thought processes invalid; therefore, to overrule decisions at this point risks contravening human rights. Many participants connected this idea with that of valid thinking, inverting the distorted thinking and conceptual benefits theme. Some participants used terms such as “mad” almost ironically, suggesting that decisions made when unwell might be contrary to an individual's best interests, but still valid and worthy of respect. Others presented self-binding directives as threatening autonomy, self-respect, and civil liberties, in direct opposition to those who endorsed them as having the potential to enhance these very values. An example response from the rejection group was: “Being unwell does not necessarily mean that you are unable to make a decision that is not right for you. Considering what is best for someone is subjective and it would be against one's human rights to not allow someone to change their mind about what is happening in a specific situation when a plan is made on what to do in a hypothetical one.”

The small number of participants who rejected the use of self-binding directives on the basis of concerns about potential practical harms (subtheme) provided a contrast to those who gave responses about the conceptual harm (subtheme), insofar as most supported clinical judgment taking precedence when a person was unwell. These participants worried that treatment instructions in self-binding directives might be misguided, inappropriate for the circumstances, or even directly harmful, and might then hamper clinical decision making. Similar concerns were found within the responses of participants who were ambivalent about the use of self-binding directives; in their responses, concerns about practical harm marginally outweighed concerns about conceptual harm. Nevertheless, one response clearly questioned psychiatric viewpoints and rejected self-binding directives as potentially removing the benefits of mania. A typical example from the rejection group about the practical harms subtheme was: “I trust clinicians to be bound by their duty to treat me according to the immediate and presenting symptoms, not some guess made the previous year. They medicate, care, and where possible seek insight from friends and family. Sad fact, bipolar disorder can smash up the world and leave one picking up the pieces at a later time. So too can a broken back or shattered knees. Help me face the daunting haul back to autonomy following an episode, not shackle and fetter me with red tape.”

## Discussion

People with bipolar disorder gave clear, rich, and varied responses describing their opinions regarding the use of self-binding directives. This empirical evidence supports the view that mental health law and practice should be adapted to accommodate a feasible self-binding directive model^1^, ^2^, ^3^ and, more generally, that we cannot define the successful outcomes of advance decision making in mental health purely in terms of avoidance of involuntary and inpatient treatment.^12^, ^16^ Yet, despite overall predominance of endorsement of the use of self-binding directives, the presence of clear rejections and ambivalence was based on varied concerns, such as logistics, or endangering autonomy, capacity, and clinical judgment, emphasising the need to take individual values and opinions into account. Such rejections and ambivalence certainly show that endorsement of the use of self-binding directives is not unanimous among people with bipolar disorder and that there are concerns that need to be addressed.

Self-binding directives are conventionally justified in terms of minimising risk by ensuring swifter access to treatment.^1^, ^2^, ^3^, ^5^, ^17^, ^18^, ^19^ However, the dominant justification given for the use of self-binding directives was the theme of distorted thinking when unwell and the resultant impairments of decision-making abilities, rather than risk avoidance. This dominance is all the more striking, given that all survey participants resided in countries where mental health laws are based on assessment of mental disorder and risk, rather than decision-making capacity. This finding might provide support for the enactment of so-called fusion proposals, in which mental health laws are based on impairments of decision-making capacity, rather than risk.^20^ Some statements supporting the use of self-binding directives did present minimisation of risk as a justification, particularly suicide risk. The possibility of such risk reduction might be a powerful argument for the use of self-binding directives, given that it is estimated that “about one-third to one-half of bipolar patients attempt suicide at least once in their lifetime and approximately 15–20% die due to suicide”.^21^

Participants presented distorted thinking when unwell as a determinate, unwelcome, and uncontrollable shift from healthy, authentic, and rational cognitive processes of decision making. This description contrasts with the commonly expressed fear that a self-binding directive might be implemented before the individual's decision-making capacity for treatment is sufficiently impaired. This fear has been a primary factor hindering clinical and legal introduction of self-binding directives, insofar as it has led to either rejection of self-binding directives or the imposition of impracticable legal safeguards.^1^, ^2^, ^3^ Survey responses within the distorted thinking theme appeared to indicate widespread confidence that the transition to a severe state of illness would be accompanied by clear impairment of decision-making abilities concerning treatment. Concerns about early implementation of self-binding directives were presented as a reason for overall ambivalence or rejection of their use in only six responses.

The survey responses also challenge various alternative models of self-binding directives that have been proposed by researchers to address this early implementation problem and have been summarised by Gergel and Owen.^1^ Most controversial is a competence insensitivity model, that would allow implementation of a self-binding directive regardless of the individual's decision-making capacity for treatment.^1^ Other models de-emphasise decision-making capacity in various ways. Some models view the self-binding directive as prioritising an individual's long-term values over those they hold when unwell;^1^ some propose risk, not decision-making capacity, as the criterion for implementation;^22^ and some adopt the idea that decision-making capacity varies by degrees, rather than there being a determinate threshold for implementation.^19^ By contrast, most responses in our survey from service users with bipolar disorder, whether they individually endorsed or rejected the use of self-binding directives, appeared to assume that impaired decision-making capacity for treatment was a prerequisite and the reason for implementation of a self-binding directive. Moreover, most of these responses appeared to imply the acceptance of the medicolegal notion of decision-making capacity as a threshold concept, when they presented distorted thinking as a distinct, determinate shift from healthy and unimpaired decision making.

Concerns about vulnerability, often raised with respect to self-binding directives,^1^, ^2^ do not feature in the survey responses. Such concerns might reflect status-based assumptions about the inherent psychological and epistemic vulnerabilities of people with mental illness and intellectual disabilities by those without lived experience of such conditions.^23^, ^24^ For instance, respondents did not express any concerns about potential emotional distress from discussing disturbing memories and topics while drafting a self-binding directive, and concerns about undue influence, or about self-binding directives hampering clinical judgment, were raised in only 12 responses. Respondents appeared ready and able to articulate details about their illness and its effects on their thinking and decision making, and to understand these effects with great insight and clarity. Similar findings about the inapplicability of such concerns about vulnerability were reached in a more general study of advance decision making in mental health,^8^ and in studies suggesting that people with bipolar disorder tend to have good insight and understanding of illness when in remission.^25^, ^26^ The degree of understanding shown in responses in our survey challenges the view that poor insight, or inability or refusal to acknowledge and understand one's condition, and even lack of decision-making capacity for treatment, persist during remission in bipolar disorder, and could impede shared decision making.^27^, ^28^ Such a view is reflected, for example, in a Louisiana, USA, law requiring decision-making capacity to be formally certified by a clinician when a service user drafts an advance directive for treatment of mental, but not physical, health.^2^

Many survey respondents appeared to accept and articulate, either explicitly or implicitly, the experience of impaired decision-making capacity for treatment during severe episodes of bipolar disorder, with many including other psychiatric diagnostic categories and concepts sometimes considered controversial, including psychosis, delusions, and loss of insight. These respondents presented these experiences as constituting justifications for others to impose treatment decisions that can override their own treatment decisions while severely unwell. Such views could be seen to challenge human rights-based rejection of mental capacity assessment and involuntary treatment, and to help address some of the difficulties associated with the need to provide protection, while also respecting agency and autonomy. In particular, the UN Committee on the Rights of Persons with Disabilities has, in its General Comment No 1 (GC1), called for the abolition of both mental capacity assessment and involuntary treatment.^10^ However, the Committee also clearly supports advance decision making in its GC1, stating “all persons with disabilities have the right to engage in advance planning”,^10^ which has led countries such as India^29^ and Australia (in some states and territories including the Australian Capital Territory)^30^ to incorporate advance decision making into their mental health laws.^3^, ^31^ In its commitment to promoting “individual autonomy”, the UN Committee on the Rights of Persons with Disabilities presents mental capacity, defined as “the decision-making skills of a person”, as a flawed, “highly controversial” concept, which is “contingent on social and political contexts”.^10^ Furthermore, it argues that to deny any type of legal capacity, on the basis of impaired decision-making capacity, is inherently discriminatory and contravenes the individual's core right to “equal recognition before the law”.^10^

The position of the UN Committee on the Rights of Persons with Disabilities would seem to remove the medicolegal foundations for both self-binding directives and advance decision making in general.^32^ More broadly, disagreements over whether coercive care can ever be justified have led to an impasse in the UN human rights system.^33^ The results from our survey might help to circumvent such difficulties, if we view self-binding directives as a means to support, rather than prohibit, the exercise of legal capacity. Clearly, many service users with bipolar disorder have found that severe illness distorts their thinking and thereby renders them temporarily unable to exercise legal agency and autonomy. They wish, therefore, to exercise their autonomy and manage their condition through advance treatment decisions that can be followed during future severe episodes.

A limitation of our study is generalisability, given that the survey was available only in English and the participants were predominantly white British, female, and well educated. The use of the UK mailing list as the sampling frame and a low response rate introduced further selection bias, as people with a previous awareness of or interest in advance decision making are likely to be over-represented and the survey was only accessible to those with the necessary digital resources.^11^ Additionally, there was no means for checking the self-reported professional diagnosis of bipolar disorder indicated by respondents.

The use of self-binding directives has also been discussed with regard to other types of episodic mental health condition. It would be useful to explore the idea with a broader range of participants, in a broader range of contexts and disorders. An implementation study (REC number 19/LO/1142) on self-binding directives for people with bipolar disorder, led by LS, and being implemented by LS, TG, GO, LR, and ARK, uses purposive sampling and actively attempts to engage those who have experience of compulsory treatment. Participants have been recruited to this study from a range of clinical services and might be less actively involved in third sector groups, such as Bipolar UK, and include a broader demographical range.

The most striking aspect of our results is the number and variety of endorsements by service users with bipolar disorder justifying the use of self-binding directives on the grounds of a major determinate shift and distortion of thinking and decision-making abilities occurring when unwell. The ethical, policy, and practical implications of these findings for advance decision making and decision-making capacity need further exploration. TG has been working on a more detailed analysis of answers from the distorted thinking theme, and further interviewing of service users with bipolar disorder or other mental health conditions could be valuable. The variety of responses suggests a need for a more personalised understanding of decision-making capacity,^1^, ^16^ a particularly important point given the increasing significance of capacity assessment in mental health law and practice. Although the focus of the current study was on service user responses, TG and PD also analysed the dataset of 110 free-text carer responses, finding quite a strong association with the service user results. It would be valuable to consider these results in more detail, to obtain perspectives from another key group of stakeholders.

To conclude, these results highlight both a need to recognise the rights of people with bipolar disorder who want to use self-binding directives to manage their health and advance their autonomy, and the difficulties of trying to find a single approach to maximising autonomy within human rights in psychiatry.

## Data sharing

All survey participants gave permission for their anonymised answers, including quotations, to be used in publications. We have therefore published the full dataset as an appendix in this publication.

## Declaration of interests

We declare no competing interests.
