# Supplementary material for: Reasons for endorsing or rejecting self-binding directives in bipolar disorder: a qualitative study of survey responses from UK service users
Source: Lancet Psychiatry. 2021 Jul;8(7):599–609. doi: 10.1016/S2215-0366(21)00115-2 (PMC8211565; doi:10.1016/S2215-0366(21)00115-2)
Supplement: Supplementary appendix [file mmc1.pdf]

THE LANCET
Psychiatry

Supplementary appendix

This appendix formed part of the original submission and has been peer reviewed. We post it as supplied by the authors.

Supplement to: Gergel T, Das P, Owen G, et al. Reasons for endorsing or rejecting self-binding directives in bipolar disorder: a qualitative study of survey responses from UK service users. *Lancet Psychiatry* 2021; published online May 20. http://dx.doi.org/10.1016/S2215-0366(21)00115-2.

Appendix

Full dataset of responses

Q1 = Some people think a “self-binding statement” is a good idea. This states that the person wants the contents of their advance care plan to be respected even if they no longer agree with it during an episode of illness.

Q2 = Why do you think this?

Classification as: Y (Yes/Endorsement); A (Ambivalent); N (No/Rejection)

Q1	Q2	Classification
1	Unwell is unwell.	Y
1	Due to psychosis.	Y
1	It's based on capability	Y
1	I do think proper assessment to write this needs to be checked over time to see when I am well enough to do this - I am more likely to come out with idea's in manic mode and seemingly well - when I am well I doubt I'd bother	Y
1	When manic I might not make wise decisions	Y
1	If I were to be in a manic or depressive episode, I would not deem my decisions at that time to be informed decisions/informed consent.	Y
1	I would want to follow my plan that I made when mentally stable since in the midst of either depression ,or mania things are not always as they seem to be.	Y
1	Sometimes, the manic state is too advanced for rational and sensible decisions to be made by the patient.	Y
1	Because the decisions would be made during a time of sound judgment	Y
1	Because psychosis intrinsically, temporarily, alters one's judgement.	Y
1	You can not think straight when you are poorly,	Y
1	During depressive period, I am not able to decide properly.	Y
1	Because they were my wishes when in sound mind.	Y
1	When you are unwell you are not thinking rationally and what you state you want might not always be your true wishes.	Y
1	Mania alters thought processes.	Y
1	When you are stable it's easy to know what you need ,when you're I'll you can think or see clearly enough to know you need to help you	Y
1	Because the decisions I have made when healthy and relatively safely with my care coordinator and my family are my true voice, if I lost any of my usual capacity I would likely be making very unwise decisions and then I would expect that my WRAP and the member of my family that I have elected to take control of my meds etc to be the voices that are respected.	Y
1	When Psychotic or manic or depressed you can become another person & irrational.It is easy to make bad decisions when ill,that may not be in my best interest. You cannot have the lunatics running the asylum?Can we?	Y
1	Because when unwell I don't always have my own best interests at heart	Y
1	in a manic state often the decisions made previously are distorted by florid thoughts, a health care professional reading the `self-binding statement` to the patient would help to bring some sense to the chaos	Y
1	From my memory, I always take the advice of the staff in the section ward but if my delusions in mania affected my ability to see right from wrong, I may hinder my process to get better.	Y
1	Because of there state of mind. They are not stable at making decisions	Y
1	It is a question of Reality. We may all have our own realities within which we are able to function. However once we loose the ability to adapt and function within society we are also loosing touch with a true Reality. No "man" is an island.... We are all products of an environment..... If we cannot function and interact within that environment what is the purpose ???	Y
1	when I am manic I am not "myself", my judgement is not only impaired, it is destroyed.	Y

1	When I am unwell I am unable to make sensible decisions and frequently change my mind. A self binding statement when I am well would clarify my constant indecision.	Y
1	The plan would have been compiled when well, thinking of ones best interests if thinking becomes disordered at a future time when unwell.	Y
1	Simply because I am bipolar, making a plan when manic or depressed would be crazy, so anything decided when I'm rational must be respected	Y
1	Because losing insight into one's illness and experiencing resistance to treatment are such key features of bipolar episodes for so many people, and lack of treatment at these points can lead to harmful behaviours if treatment is not imposed.	Y
1	Because you're definitely not 'yourself' when manic and don't necessarily behave rationally. For example one time I was very anti doctors and very anti men - sinwouldnt co-operate, whereas in my 'right mind' I would be keen to do so and have the best treatment.	Y
1	Because when a person becomes well again they would regret their initial plan has been changed as when unwell it is impossible to rationalise competently	Y
1	Because when I am unwell I will not agree with my advanced directives	Y
1	Whilst unwell it is very common to have ideas that are not in one's best interest.	Y
1	The statement should have been made when the person was well and hopefully rational.	Y
1	Because when I become unwell I do not accept that I am unwell as I only become manic I feel amazing and do not always accept that I am ill	Y
1	When you are unwell either Manic or very Depressed you do not see the world as it is. Providing the plan was made & kept up to date when you are well it is more likely to be what you really want when you get back to thinking straight.	Y
1	Judgement impaired when unwell.	Y
1	I never agree with being in hospital, being seen by the crisis team etc when I am even slightly unwell. I loose insight quickly and completely.	Y
1	Because the whole reason for the care plan is that you will be unable to accurately communicate your needs when unwell. This includes not being able to think lucidly or make informed decisions. Therefore if you are able to override the plan while mentally unwell you could make bad or even dangerous decisions. This would mean making the plan itself is utterly pointless.	Y
1	if you are unwell you cannot always make rational decisions	Y
1	You need protecting from yourself with this illness. This also protects family from suffering.	Y
1	When acutely unwell, loss of insight in to your own condition and medical treatment needs can occur. If I had a well planned, researched and considered plan that I was satisfied with when well, then I would be happy for what I would see as my informed and insightful "well self" being listened to above my "acutely unwell" self.	Y
1	Basically I would refuse something I would have considered rationally when I am unwell.	Y
1	If one is extremely unwell in a 'crisis' then thoughts and feelings could change significantly from 'well state'	Y
1	It is created in advance whilst considered to be of good mental state, what would be the point if you can overide this carefully contructed, well informed, action plan if its likely to be overthrown whilst in altered mindset. However, is logical thoughts contextualize your thought process and decision to contest original plan, then this should be considered by treating specialist.	y
1	Because when I am unwell my view of situations is often very different and unrealistic	Y
1	During Manic episodes it is impossible to make the right decisions about treatment.	Y
1	As many decisions about treatment as possible should be made when you are of sound mind.	Y
1	when manic i lose sight of what is good for me	Y
1	It's a no brainer. So important to prepare for such an event to receive the treatment I would want.	Y
1	The statement is written when you are relatively well and have reasonable perspective on all facets of life. To be allowed to change things when moods are out of balance and perspective is lost is potentially disastrous.	Y
1	Because the plan will be made when I am well and rational and should I be ill with mania or depression I will be in a totally different headspace	Y
1	Because it is best to stick with decisions made when you are well and have a better perspective.	Y

1	We should have rights too . As we made it before we fall unwell	Y
1	When unwell you usually don't have the ability to think sensibly, so best stick to something you agreed when you were in a better state of mind	Y
1	Because I cannot tell when I need help.	Y
1	I have in the past refuses ECT when I should have accepted.	Y
1	I cannot at times make sensible decision and do the opposite of what I'm being given help with	Y
1	Sometimes the disorder distorts your thinking and paranoia can set in so your need your rational, stable thoughts to guide you.	Y
1	When I'm manic I tend to think I can manage without drugs, when they are in fact helpful.	Y
1	Irrational when unwell. Can present as lucid but am not. Up take stupid risks and bad decisions up or down.	Y
1	because sometimes when I am not well I do not even know who I am, let alone what I should/should not wish to do.	Y
1	The plan is written when you are feeling well and mentally sound of mind. Decision making is more rational.	Y
1	Mental state is not what it would usually be and would likely vary from what you would want outside of mental ill health.	Y
1	Bipolar manipulates your decision making abilities so it is important to have a clear plan in place that was written when the service user is of level mind and informed correctly.	Y
1	When acutely unwell it is hard to see wood from trees but if the document was written by myself when well I would have looked into all options available and made an informed choice.	Y
1	Sometimes I am more unwell than I realise myself	Y
1	During an episode, I do not have clarity of thought.	Y
1	Because I tend to have better insight and judgement when I am stable compared to when I become Hypomanic, plus although it may be difficult for me to hear - I devised this plan for myself!!!.	Y
1	Because it is possible that my judgement is negatively impacted during a manic episode.	Y
1	At the time of writing the plan you would be well, so making those decisions would be done in the right frame of mind	Y
1	Your mind isn't in a capacity to make decisions	Y
1	As long as self-binding statement is up to date.	Y
1	When unwell it is likely that one loses touch with reality - becomes suspicious of others and interprets black as white and vice verse	Y
1	Because wishes of the person involved should always be respected	Y
1	I don't know what my feelings would be when ill. And I often get very strange ideas when ill (for instance delusions of granduer). So what I decided when well is probably a more sensible option.	Y
1	Because when I am unwell I am irrational and unable to make healthy or safe decisions	Y
1	You are unwell and lack capacity. I would agree with this statement looking back at when I was last sectioned but my views were vet different at the time due to my illness. It is my well views and opinions that should be acted upon.	Y
1	Being unwell changes thought processes, our brains lie to us. If a statement has been made when well then hopefully balanced decisions were made	Y
1	If you have a psychosis you don't know what's best for you	y
1	Because I understand that this would be in place as I made it when I was of sound mind and I would want to be treated in a way which would help me get well in spite of any resistance I might otherwise put up.	Y
1	Because when your very unwell your thinking can be so deluded you might just want to do or try anything that when you where well you thought would not be wise.	Y
1	Because when I'm truely unwell I can't make a decision and I'm less likely to agree with what I think when I'm stable as I suffer mainly from high episodes	Y
1	Because when I am unwell good decision making is not usually what I manage, if anything bad decisions are what I make when unwell	Y

1	When I am well and to some extent when I am "on the way down I am able to understand and acknowledge that the feelings will likely pass. However, in my most depressive phases I cannot comprehend this so can be adamant that nothing will change this so am reluctant to accept help. Similarly, when I am up (though fortunately, these days, I don't have full blown manias) I don't always take my meds as regularly as I should as I either forget or don't feel I need them. When I am well, I know they are important.	Y
1	If it is written when you are well it is what you want when your well. When your unwell your more likely to disagree or make unwise choices. If it is explained this is what you wanted to happen. For me I would like my husband to have my bank and credit cards and to give me cash. However, when I am manic I would not like this, but I know it is a way to prevent me from spending.	Y
1	Because when we are unwell we make rash decisions which not always make sense or at the time be in our best interests.	Y
1	When you are unwell your thinking can be confused and not in my best interest	Y
1	you have to rely on the 'well' you	Y
1	The Plan would have been drawn up during a period of stability	Y
1	Gives people voices before they are ill and unable to make them.	Y
1	State of mind can fluctuate wildly during manic/depressive episodes	Y
1	In a manic episode the thoughts are not rational	Y
1	Because the advance care statement should be made when feeling well. It is difficult to make good decisions when unwell.	Y
1	When unwell you are ill and cannot see reason,so a statement which is made when you well and able to voice an opinion is vital. After all we all want to get better and feel safe and well. My worry is that the statement of self care is over looked and deviated from.I tend to worry about this alot.	Y
1	When I am manic or hypomanic I think I am not ill unless certain symptoms appear. I realise after an episode that these specific symptoms have not occurred always but I was still ill. In feeling 'well' I could refuse treatment or care when I really need intervention. When I am depressed I can feel too tired to fight to get better and want to be left alone to get worse on purpose so I can then end the pain	Y
1	I wouldn't have the capacity to make careful, reasoned judgements when unwell	Y
1	When in crisis I am not always able to make considered decisions, particularly relating to medication. I would like to have considered this at a point when I am well and rational.	Y
1	When I am ill, I am not of the right mind. So making a decision at this time would probably not have a good out come.	Y
1	You are looking out for your I’ll self when you are well because you can’t advocate for yourself when you are ill	Y
1	As when having any type of episode self conscious decisions aren’t exactly a strong point	Y
1	When I’m ill I often don’t have my best interests at heart	Y
1	Being well and not being well are quite different states. Being not well puts one in a tricky place for making good decisions.	Y
1	Because when I am unwell I think and act very differently to when I am well. When I am unwell I am unable to make positive, healthy, rational decisions about my own care. When I am well, I have quite good insight into my illness and how best to manage it.	Y
1	When unwell a lack of insight prevents me from making decisions.	Y
1	Because there is a point that I will arrive at where I think I know everything. At that point, I need someone to take over.	Y
1	The advance care plan has been written when not ill and states how you wish to be treated. When ill you may not agree with this however I feel it should still be followed as it would be in my best interests and hopefully prevent relapse or a period of long illness.	Y
1	when manic, panic and paranoia can take over and we aren't always the best judge of our own condition. To have people who know us well, ie GP,Family member is vital.	Y
1	When I am unwell I act and think very differently to when I am well.	Y
1	All these orders are made when in a fit state of mind. Manic, mixed phases have me believing I know what is good for me...experience showed I didn't so self binding is a great idea	Y

1	As it makes everyone around you aware of your condition and your needs	Y
1	As patient may be unable to make lucid decisions that are in his/her best interests	Y
1	As when you are unwell you do not think clearly and usually refuse all treatment. I think they should be regularly reviewed and signed by service user and professional reviewing it.	Y
1	As you don't always going straight when your unwell.	Y
1	Because I became detached from reality during my manic episode	Y
1	Because if someone is in crisis and not in a good state of mind this statement will reflect their wishes despite their statr of mind It will protect them	Y
1	Because if you are unwell you cannot think clearly	Y
1	Because in theory, or hopefully the advance care plan will have been written/put together when stable, so of sounder mind.	Y
1	Because it is the nature of the condition to make poor decisions and take risks when unwell which you wouldn't consider when well. Therefore you are almost certain to want to change the decision when unwell which defeats the object which is protecting your property and saving you embarrassment when unwell. However you should be able to change the decision when well.	Y
1	Because it shows wishes made and what I would and wouldn't want. As soon as ur in crisis people think u can't think rationally	Y
1	Because I've written it when feeling well I would have thought deeply about how I want my treatment.	Y
1	Because otherwise the plan is pointless!	Y
1	Because people can be delusional, psychotic etc and temporarily believe in courses of action that are not beneficial to their mental health.	Y
1	because then the patient can really confirm the document	Y
1	because these were my wishes when I'm well	Y
1	Because they agreed to it when they were in a better frame of mind.	Y
1	Because they were themselves when they were well - when they compiled the statement - and they are not themselves whilst they are unwell.	Y
1	Because this is made during a well period and should be respected	Y
1	Because unwell I say anything and usually don't actually mean it!	Y
1	Because when experiencing manic or depressive episode you are not attuned to reality and likely to change your mind about treatment etc. The advance care plan must be created however when a person is experiencing stability.	Y
1	Because when I am ill, I do not think rationally and do nor accept that I am ill	Y
1	Because when I am in the grip of either manic or depressive states I can be unable to control my thoughts and behaviour.	Y
1	Because when I am unwell I am unable make right choices Once I spent £5000 when unwell So I am not capable of making right choices	Y
1	because when I am unwell I dont think that I am unwell and would be highly likely to kill myself.	Y
1	Because when I am unwell I suffer from delusions and act out of character and don't make sense to other people	Y
1	Because when I am unwell, my thoughts, perceptions and desires drastically change. I can become suicidal and impulsive, making decisions or plans that I (would) later regret (if I had followed through with them). I often come out of an episode feeling extraordinarily grateful that I didn't take my own life, but when I become ill the same thoughts and plans return to me regardless and I am convinced they are my true desires. I don't want anyone to listen to me when I am unwell, I explicitly would want them to take my advance plans as my true wishes and would be mortified if they were overruled.	Y
1	Because when I become manic I also become paranoid and mistrust the people closest to me. I blame them for my having to be admitted because they cannot cope with my condition. For example, on the last occasion I was hospitalized I refused to allow any information to be passed to my husband which caused a lot of unnecessary problems.	Y
1	Because when I become unwell (especially depressed) I don’t want psychiatric intervention - I just want to die	Y
1	Because when I'm unwell.I may not be able to.	Y

1	Because when manic or depressed my view on life changes.	Y
1	because when you are unwell you do not know your own mind	Y
1	because when you are well you know what you are doing and are in the right frame of mind. When you are unwell you are none of these things.	Y
1	Because you are not yourself when you are unwell (in my experience) provided the plan is written and agreed when you are well it should be honoured	Y
1	Because you might know yourself better when you're well than when you're not	Y
1	Because your incapable in making decisions when having an episode and that's why the mental health act in there.	Y
1	Better decisions are made when you are well, so when you are unwell you may be disagreeing with something that is still a good idea for you.	Y
1	Bipolar can alter my perceptions and therefore give an untrue, or at least distorted, view of my situation.	Y
1	Can't make rational decisions while manic or depressed.	Y
1	Decisions made when lucid and rational are usually thought through and consulted with.	Y
1	Decisions made when unwell are unsound.	Y
1	Dont really know what you are doing when you are unwell	Y
1	During a manic episode I would be incapable of making rational decisions.	Y
1	During an episode unrealistic and impulsive decisions can be made.	Y
1	During episodes you lose the objectivity that is present when well. It helps if such objectivity is self-binding during periods of illness, provided the circumstances have been foreseen.	Y
1	From personal experience, I recognise that when I am unwell I am incapable of making sound judgements or safe decisions regarding my care, so I would prefer to make decisions regarding my care when in a stable state and have a crisis plan to be used by health professionals, family, and friends when I am unable to keep myself safe.	Y
1	frommy experience of episodes	Y
1	I am sure this is good if you have made the care plan when you were in a stable mood	Y
1	I am very irrational when unwell but believe I would be more inclined to listen to myself	Y
1	I can agree honestly that there are times when I'm unfit to make important decisions for myself , its at that those times that my care plan should take precedent over my immediate wishes	Y
1	I can't make lucid decisions when I am having an episode.	Y
1	I dont make the best decisions when im unwell	Y
1	I just do, I am so sorry unable to stay with the survey, I am so tired, I can come back later	Y
1	I know how i want to be treated, what will help and what would exacerbate my symptoms. When unwell i may not appreciate this or be able to convey it. I think an advance care plan would facilitate my recovery from episodes.	Y
1	I know i do not have my best interests at heart when manic.	Y
1	I know that when I am in a delusional state I need medical help and am prone to do weird and dangerous things and certainly not listen to reason.	Y
1	I think this because it has your wishes and it also helps the professionals know how you are in different situations and it's a clear picture that may help in guiding them into a plan of there own for me to go forward with.	Y
1	I think this because when I'm unwell I would not be making the right decisions for myself and my wellbeing.	Y
1	I think when you are very ill you are not in a position to make these kind of decisions about your treatment. I also think when I have been ill I have been confused, delusional and paranoid and made decisions I wouldn't when well.	Y
1	I use it myself. I give mental health staff permission to section me if needed. It gives back the power to me.	Y
1	I would only like to produce one when i was balanced and well, so yes to this.	Y
1	If compiled when a person is well it is likely to reflect the real, reasoned wishes of that person. A person may be less well reasoned and self aware and therefore less likely to make best decision for themselves when unwell. The wishes agreed when person was well should therefore carry more weight than the persons wishes when unwell.	Y
1	If I am not able to make such decisions at the time then I think this would still ensure I am being treated in a person centred way	Y

1	If I had planned a reasonable care plan and then turned against it because I was high I would expect those who care for me to ignore my manic self and refer to a document made when I was rational	Y
1	If I'm unwell I lose insight and would disagree with any plan made. Therefore self binding statement is essential.	Y
1	If I'm writing it when I'm stable and have perspective, then my requests are rational and appropriate. I won't like it during depressive/ manic episodes, but it would hopefully help me get back to a more stable place.	Y
1	If the statement has been prepared whilst person was well then it should be adhered to.	Y
1	If you & professionals have diff ideas on if you are well or not	Y
1	If you agree to it when your stable, the decisions you make when you are unwell are different	Y
1	If you are incapacitated through mental illness your thought processes are likely to be unreliable. It is better to respect the advance care planning.	Y
1	If you are well and "in your right mind" and make a logical, rational decision about your care, then you are in a much better position to make a correct and sensible decision which is in your long term interests, than when you are unwell and in an unhealthy, compromised mental state.	Y
1	If you're unwell, you're not able to make those decisions. It's a difficult one, but a self binding statement isn;t compulsory, so if you sign up to one, you're acknowledging that you're frequently likely to change your mind when unwell	Y
1	Illness prevents clear thinking.	Y
1	I'm not always rational	Y
1	Important everyone concerned aware of plan	Y
1	In mania can lose sight of how unwell I am	Y
1	It can be too hard to express what I want when I'm very unwell.	Y
1	it sets out exactly your wishes and it Has to be followed	Y
1	It support you when you aren't able to think clearly.	Y
1	It was done at a time of wellness so should be respected.	Y
1	It's my decision to make all the time I'm well I have the capacity to make that decision.	Y
1	It's the wishes of you at your best not your worst	Y
1	More control	Y
1	My choice	Y
1	No point in having one if individual can overrule it when seriously unwell	Y
1	No point making a plan if you are not going to stick with it.	Y
1	Once insight is lost so is rational thinking / decision making.	Y
1	Our perceptions change when we are unwell and sometimes want harm	Y
1	Personally I struggle and a care plan such as this would take the worry out of future episode when they do come.	Y
1	Personally speaking, I think when I'm manic that I lose the logic that I have when I'm balanced and might renege on decisions that'd I'd taken when I was more balanced.	Y
1	Potential of own poor judgement when manic or depressed	Y
1	Provided the plan was made with thought and care when the person had capacity of mind, it should be followed, even if the person no longer agrees when that capacity is no longer there, because the plan is for that exact reason, to show their wishes in that situation.	Y
1	The Care plan states the expectations and wishes of the Service User when they are well and of fit mind. To disregard this is tantamount to disregarding a contractual commitment as in the Power of Attorney.	Y
1	The plan should be madre when a person with bipolar is stable and in a position to plan for their future needs and wellbeing.	Y
1	The rational decision was made when the patient was of sound mind. It would be irrational to change this when the person was mentally unwell.	Y
1	To show how much they corroborated on the plan when in a stable state	Y
1	When 6 nurses decide to push you around, pin you to the floor, give you no option bar treatment perhaps at least if you have a legal document you might have a leg to stand on later on.	Y

1	When a person is unwell they may lose insight and act in a way that is contradictory to their usual behaviour, beliefs, and wishes. It should be possible for this to be anticipated with such a statement of wishes made when the person is well.	Y
1	When emotional state changes, decisions that are made when well may be different to those when not well.	Y
1	When experiencing a manic episode, in particular, rational thought is not possible.	Y
1	When experiencing a severe episode rationality is no longer present.	Y
1	When feeling and thinking clearly, you can agree to sensible ideas of what type of care you may need. However during an episode; manic or depressive, you don't think the same and although you agreed certain things when well, it may be very difficult to deal with these decisions as they could be traumatic when unwell, even if they are there to help you get better.	Y
1	When I am depressed I become suicidal and need to be admitted to hospital as soon as possible. I should not have any say in the matter.	Y
1	When I am Ill all I see is the pain and torment of my existence, I want to die and any easy way would be seized upon and refusal of medication which I would do just cements these ideas. If I make a care plan whilst feeling normal I know I am far happier, contented and much more rational and l know that it would be bad for me and my family if I died. This is why I believe a care plan would work. At least then there is some thing in writing that I know is a true reflection of my life rather than the warped one my brain insists is true.	Y
1	When I am manic I make poor decisions. I am drawn towards destructive behaviours and am impulsive. When I am manic my care plan is most useful. My husband will sit with me and we will discuss the behaviours on the care plan that I myself have identified as being signs of developing hypomania. It is harder to disagree when you have written it yourself! I am more likely to be cooperative with the plan eg. I relinquish any access to our bank account, give my husband by passwords for internet accounts, sometime give him my car keys. I agreed to all of these things when I was well. In the past, acting on my plan has resulted in a much quicker stabilisation of my mood. A care plan is also useful for depressive episodes as it gives my husband and CPN advice on how best to support me through these periods. But the biggest improvement has been with nipping the hypomania in the bud.	Y
1	When i am unwell, I can no longer see the end of the torment, pain, sadness, loneliness, isolation, sense of not belonging or I am completely detached , robotic like doing tasks with no feeling. I feel like I am watching T.V. But the program is my life, my day, my existence. So to disregard a plan that was made when I was less unwell would be sacrificing the duty of care owed to me for my health from those who allowed the plan to change.	Y
1	When I'm ill, I can't think for myself, my thoughts are all muddled, and I am liable to make rash or bad decisions	Y
1	When in mania, I don't believe I am ill nor need support.	Y
1	When insight is lost it would help family/friends make decisions on my behalf.	Y
1	When manic logic decreases dramatically.	Y
1	When not in a fit state it's important to maintain stability	Y
1	When people become mentally ill they are likely to cease having the clarity of mind to consider what's in their best interest, so these should be drafted when they are fully functional/ at the best of their capabilities and taken as they wishes for such a situation.	Y
1	when relapse occurs you are not capable of good decision making.	Y
1	When unwell a person is not always able to think or act as usual. The decisions made whilst well should be respected as being how they'd like to be treated in all situations.	Y
1	When unwell I'm unable to make decisions that reflect what I want when I'm well, therefore I would want a plan written when I'm well to be followed as this reflects my choices that have been made when I'm able to	Y
1	When unwell less likely to make informed decisions	Y
1	When unwell with Bi-polar it is not a good idea generally to change any life-changing decisions when in that frame of mind	Y
1	When we have an episode of high or low mood, we are not ourselves. We do not think straight.	Y

1	When well I understand my illness and am quite clear what works and what doesn’t. When unwell I can become confused frightened and make poor judgements,I have often agreed to treatment purely to get released,knowing it’s the only way to regain my liberty and freedom . Some of the treatments I’ve consented too have been extremely determental to my wellbeing. Regaining mental statbililty and wholesomeness has been long and arduous ie returning to work and driving has been prolonged due to consenting to medication I know is not helpful in the long term but will get me out of hospital. So it’s vital my wellness statement is adhered too.	Y
1	When we're going through a phase, either manic or depressed, we might not be in the right state of mind to make a sensible judgement.	Y
1	When you are unwell you lose sight of what’s important, you cannot think straight, so to help the people involved in your care it’s good to have a statement to use, as they know that’s what you want.	Y
1	When your ill you don’t realise your that I’ll.	Y
1	You can lose insight	Y
1	You cannot predict your capabilities, shoud the illness present itself.	Y
1	You can't trust your own judgement when very unwell.	Y
1	You lose the ability to see your previous rational thinking when manic.	Y
1	You’re not thinking rationally during an episode.	Y
2	The alternative seems to contradict the thinking and logic behind having an advanced care plan in the first place.	Y
2	not sure, but there is no point in making it if it is not binding	Y
2	Because the plan is a snapshot in time with the information available. If I have capacity, I may prefer a different route based on new knowledge. I would look to any executor to exercise discretion if my changed preferences appeared to be indicative of diminished capacity and if so uphold the original plan, otherwise respect my wishes at that point in time	Y
2	This is why it's been stated in the first place	Y
2	Like saying you don't want an epidural when pregnant but when faced with reality of pain may be begging for one so may not not how you feel until you face a certain issue	Y
2	When I'm manic, I'd absolutely ditch my advance care plan, because I would think that "obviously I don't need it". Defeats the purpose of an advance care plan.	Y
2	I think even if when you are well you decide something if you want something different when ill your choices then should be considered too. It could be really hard & distressing when unwell if mental health professionals were insensitive (as some can be in these situations) and said ‘you yourself agreed this should happen.’ I don’t know what that would do to you psychologically. If at the time you didn’t want it you could feel that not just the doctors but your own former self has it in for you. So yes it should inform but should be handled sensitively which I’m not convinced the staff I’ve met in psychiatric hospitals would do. I suspect an advance directive would only be used when they agreed with it. If they didn’t the mental health act powers would trump it.	Y
2	Because your opinions could change	Y
2	Because you never know what circumstances could arise	Y
2	Might not be up-to-date and circumstances may have changed. Cannot predict the situation or nature of episode so hard to be certain of what care you will want/need. Can only really be used as a guide but shouldn’t be fixed as we can’t predict what will happen so can’t outline all the specifics.	Y
2	With so much to think about and deal with, people might forget to update it. A re-visiting of the plan every year would be very important, or some other way of reminding service users to keep the plan up to date.	Y
2	Maybe you are too sick..	Y
2	If it isn't self-binding, then there isn't much point of having it in the first place.If it has been made when they are well/ in a sound mind, it should be stuck to. The fact that they might disagree with something they thought when they were well might show that they are currently unwell.	Y
2	Relationships can deteriorate remarkably quickly under the stress of a partner going into mania.	Y
2	Since the statement was made when the patient was well, and probably in collaboration with medical professionals and carers, it is unlikely to be of any harm, and referring it will help the patient to reestablish continuity in their lives.	Y

2	When manic you are not rational however the way you are treated has a massive impact on you and your ability to recover	Y
2	as these were the wishes made when the person was of sound mind	Y
2	When I have been unwell I simply do not have capacity to make sensible judgements or decisions. It would be the role of my family with my psychiatrist to explain to me that I have already made my decisions about my care . I realise that were I to become obstructive or violent / abusive then someone has to take responsibility for my treatment and my advance care plan is the basis for this.	Y
2	When we are very unwell with mania or depression our judgement and capacity may well be impaired. I would rather that treatment decisions were made based upon my judgement when I am most healthy and well.	Y
2	As they were of sound mind when deciding and know what outcome will be.	Y
2	The whole point is that when unwell rational thought is disrupted so the purpose of the plan is to record your views when rational. Its because our thoughts change when unwell that a care plan is made. It defeats the purpose to ignore the plan when irrational thoughts are prevelant.	Y
2	Because when in a vulnerable position sometimes you do not make the best decisions.	Y
2	Because,when well they made the decision with conscious consideration and know what's right for them	Y
2	I have been too manic to make these decisions and will get rebellious and make rash decisions. I'm not sure I'd even be able to handle the detail of it, but would certainly prefer that it was adhered to as I had taken time when relatively well to think about these things. My advocate being aware of the binding statement. Certainly the only person I have trusted when in hospital. I can get really paranoid.	Y
2	Because how you view things can change when unwell due to your illness, particularly when manic or mixed	Y
2	If you can argue rationally if there is a particular reason for something to change then it should be considered. Even when you are ill you should have as much agency as is possible to give you. But I think the care plan should take precedence in most cases.	Y
2	I don't always have insight into what I need when Im ill so it would be good to commit to a course of action I helped design when well. On the other hand there needs to be some flexibility because a crisis plan doesn't always cover all eventualities and what works one time might not always work.	Y
2	Tricky. It is possible to see the argument against in terms of freedom to change one's mind. The person him/herself can think it through and decide at the outset, without pressure by others.	Y
2	Because when you are ill you loose control and someone else really has to make the decisions	Y
2	I lie and pretend I'm well when I am unwell which I don't realize till I'm better. It is so scary.	Y
2	If it has been planned during well periods then that is a true representation of wishes.	Y
2	Obviously when manic or depressed you may no longer be of reasonable mind, or able to make logical decisions. It would be helpful to have a plan, and have that plan stuck to. When sectioned, all this is moot. However, if it's something that may cause permanent problems, I would like a say whether I was second or not - if it was on my statement. Eg. I know ect works and is much safer than it used to be, but I would refuse it for risk of losing memories, even if that risk is slight.	Y
2	Hopefully you werent too ill when you made the care plan	Y
2	If written in a stable mind, then it should stand if mood is unstable.	Y
2	People vary widely and there is no right answer to this. Obviously it can be included in any advance care plan. My own feeling is that, ex hypothesi, judgment is likely to be impaired when others consider someone mentally unwell. There is another reason, which is that some people (here I include myself) become vulnerable, over-compliant and suggestible when unwell, and later regret agreeing to some suggested treatment. The third reason is that recognition that judgment can become impaired, implicit in the idea of 'self-binding statement' , is ipso facto therapeutic and prophylactic. The idea itself is as old as Homeric epic: Odysseus used the technique in order to listen to the Sirens, by ordering his men to block their own ears, tie him to the mast of his ship and to ignore instructions to untie him until they had rowed well clear. Extreme mood swings have a similar addictive quality. That said, other people may find the idea too challenging to contemplate and it may well be a mistake in some cases to suggest it, and certainly a mistake to pressurize people to make one.	Y
2	When ok i can appreciate that hospitalization might be necessary in certain circumstances which i will not agree with if unwell	Y

2	An advanced care plan is most likely to be produced when the Bipolar individual is more stable and has support and dialogue with the carer(s), family and friends. Bipolar individuals during an acute manic or depressive phase may not be capable of making decisions in their own best interest.	Y
2	Because if I'm in a bad space I will not want to do things to help. When I am balanced I think I will resent this but I don't when I am well. So I would know that taking time out is what I need to do ... But when I am ill up or down, I will refuse.	Y
2	When you are well you can think for yourself. When you are unwell you are more likely to be paranoid if you are told others are making decisions for you.	Y
2	From personal experience I know that I become irrational when very unwell. My main reason for having an advance care plan would be to ensure that my rational decisions, made when well, can be enacted.	Y
2	The plan should have been made when the person is well, no decision taken when unwell	Y
2	I'm conscious of my own lack of rationality in mood extremes. Having a process to change a document would be better than trying to do it on the fly. At the same time, circumstances can change quickly so some flexibility should be allowed - both for professionals involved in making decisions in my best interests and for myself in changing mind.	Y
2	Due to manic nature of illness clarity of mind is broken.	Y
2	Medical matters such as physical should also be included, often people, I feel neglect there physical health whilst they are unwell they may not be aware that they have a condition. My brother had a serious illness whilst in a psychiatric hospital. It was not treated. He was admitted to A and E this made his mental health worse. Recovery was slower.	Y
2	I know that I am not myself when I am unwell. As such, decisions I made for my own well-being would be more important than decisions I make when unwell. It stands to reason that I would make better choices before the fact. Sadly, there are one or two issues. The first is that I might have changed my mind and never updated the plan. The second is that practitioners may interpret certain instructions or preferences to suit their own needs and preferences. Neither one is insurmountable (they just require a clear plan which can be updated easily), but it still makes me a little hesitant.	Y
2	I think insight and understanding of the self is stronger and carries more weight when psychologically well and stable and provides a path back when the mind becomes unstable, where navigation becomes far more difficult and be detrimental to the mind's equilibrium. E.g. withdrawal and staticness in depression and things moving too fast in mania.	Y
2	Very difficult question to answer! I think as they wrote the self binding statement with a sound mind, they would have been able to make the best judgement on what treatment suits them during an episode.	Y
2	I trust my decisions when I am stable.	Y
2	Because things change so much when episodes really go to hell.	Y
2	When you write it you have mental capacity.Also hopefully agreed and signed with a health care professional.When you are unwell you mostly think you are well or don't recognise how unwell you are.	Y
2	Personally, the decisions I made while I was unwell are generally not that reasonable/sensible, and should not be followed.	Y
2	As an individual with bipolar, I am well aware of my condition yet I cannot honestly say I have made all the right decisions about my treatment or my wishes along the way. It isn't a case of "blaming" the bipolar, but sometimes the underlying fear and 'what if' games that mental illnesses can play on an individual don't always let you have the most objective or practical view. It's easier to establish a 'comfort zone' that makes you feel reassured than necessarily take the harder steps. I have been very lucky that I have had both medical professionals as well as family to support me and sometimes make me go through with treatments etc. even though my own reaction would have been to shy away at the time it mattered most. I do believe there is still some need to feel you do of course, have the ultimate say over how you are treated as an individual, but I definitely think there should be at least some intervention or wider discussion with all parties involved before an individual can just effectively 'opt out' of their plans when something changes.	Y
2	I made the plan when I was well	Y
2	Given the nature of the mindset you have when you are unwell, there is a high level of imcapability to make decisions. I think it is more sound to have had something written up in advance when the mind is lucid and clear on the instructions made.	Y

2	When a person is unwell they might not be thinking rationally. However that is not always the case	Y
2	They may not know what is the best way to get themselves better. They can express wishes such should be followed as best as possible.	Y
2	Because when I'm in a depressive state I want to die, when I'm not I don't. I'm a different person with different thoughts, feelings and reactions when I'm depressed or even manic, it wouldn't be fair if I'd stated in my care plan to persist in treatment to get better but was refused because of my depressed or manic state. More nurses, gps or nhs staff need better training in recognisig traits of bipolar or even being more understanding. I've tried to hide things or talk bout things from/with nurses or gps because I don't feel they understand the respect me.	Y
2	Probably have a better idea to do the plan while mentally more stable to make better decisions.	Y
2	Because presumably the person has been in normal/balanced state when they created their advance care plan.	Y
2	Unable to think clearly.	Y
2	When I am unwell I cannot think straight and might not be cooperative.	Y
2	If I was unwell/psychotic I would request that my wishes when I was mentally competent be respected. I would not be able to make a sound decision when unwell.	Y
2	When we are unwell we do not have a right perspective. That's why it needs to be done when we are well	Y
2	A person going through a manic episode has, by definition, seriously distorted thinking. If a person is manic to the extent that they cannot make their own decisions then again, by definition, they are not in a right mind to refute their advance care plan - otherwise it's basically worthless.	Y
2	Yes because the plan is in place for when I am unwell	Y
2	When the document was last refreshed should be considered in light of accepted on-going treatment which contradicts an out of date plan however given the symptoms of episodes it is likely that someone in any kind of episode would potentially request different treatment out of desperation or detachment from reality.	Y
2	As long as this care plan is up to date, and lets say recently revised and signed every 6 months or so, then yes I think this a could idea as thoughts, feelings and intentions during a severe manic or depressive episode can not really be relied on for the best interest of the sufferer.	Y
2	It is possible that my ideas about treatment may have changed since the advance care plan was drawn up. Therefore probably important that I was first assessed in a crisis by a psychiatrist to see if I was able to make a rational altered decision about treatment at that time and if not i.e. if I were psychotic, then the psychiatrist should be free to implement the previously drawn up advance care plan.	Y
2	My experience is that your judgement can be very impaired when you are unwell, and well thought out arrangements made when you are stable should take precedence.	Y
2	If you become unwell in another part of the country or abroad the psychiatric services there may use completely different drugs to those you normally have I think they should be made aware of what you are accustomed to taking	Y
2	Because at the moment there is nothing in place	Y
2	The purpose of a "self-binding" statement is to have a care plan to use when the individual is not well and does not have mental capacity to make such decisions. If the individual is not well, they are unfit to make such decisions; the care plan has been created for this purpose.	Y
2	I don't think rationally when unwell	Y
2	in case I deteriorate in the future	Y
2	During mania or depression I would make decisions that would not be a reflection of my wishes when well.	Y
2	Because if it's written at a time when someone has good insight, they're more likely to know what's best for them. I also think that they should be regularly reviewed and amended if need be to reflect changes in wishes	Y
2	Because decision making can become clouded when very unwell and you may be acting against your own interests as recognised when you were well and thinking clearly.	Y
2	My ability to make decisions is poor and my interpersonal communication falls apart.	Y
2	because you can't always think straight when you are unwell but if you made the plan when you were well this is most likely what you would really like to happen	Y
2	When you're manic you lose reasoning and a plan developed when you're well is probably a good one	Y
2	depends whether the person has been sectioned in the past. If so I would say definitely yes	Y
2	Very irrational when unwell.	Y

2	People have a right to change there mind but when Ill can not always make informed decisions and easily swayed. So it is good to make a clear decision when well that is somewhat binding, however some room to change mind if needed to.	Y
2	If it is created during a time when a person is well then the plan should be stuck to. As they often have different ideas when unwell.	Y
2	Because when manic or depressed one tends to lose perspective,	Y
2	Because almost by definition, when I'm in a hypomanic or manic episode I will resist all attempts to moderate or curtail extremes of behaviour.	Y
2	I can become very stubborn/irrational when unwell. Would not want to fall out with family/psychiatrist. Overall better than not I think!	Y
2	Because a person in crisis is not thinking as they normally would when well and when they become well again would probably reaffirm what the had laid down in the plan	Y
2	It has its problems. But it also has benefits. It's a tricky one.	Y
2	When I am manic I make very risky decisions and become completely different to my usual self. The reason I want a plan is that I would probably refuse treatment and also have strange ideas that may lead me to seek treatments I wouldn't usually want. However I also worry that I may change my mind for a legitimate reason and then be forced to comply with my plan so I am unsure about this. I feel the best solution for me is to give some of these decisions to my partner who would be able to discern if I am making the decision based on normal reasoning or not.	Y
2	People change over time, and a plan may become obsolete but be forgotten about, and not be updated to include new wishes	Y
2	Because of the difficult nature of Bipolar and it's effects on personality, thought processes, interpretation of memory etc.	Y
2	It needs to be an agreed plan but provided there is capacity then an individual should have the right to adjust it depending on their circumstances and in conjunction with their clinical team and recommendations.	Y
2	There may be occasions when I've lost insight due to psychosis. I have worked hard on developing a trusting relationship with my health professionals and would trust their judgement.	Y
2	Because when someone is ill, they may not be thinking in a logical way. They might not have capacity to decide what is best with regard to care and treatment. The advanced care plan would have been written when the patient is of sound mind, and therefore more likely to reflect the patient's true wishes.	Y
2	Bipolar can cause serious delusional episodes and a self binding statement may help the person get what they actually want	Y
2	My perceptions of things when I'm unwell can be very different to when I'm well. Assuming the care plan was put together while in good health, it should be followed regardless of whether or not I feel the same way while unwell.	Y
2	My perceptions are likely to be different if I am unwell and my judgement may be impaired at times. When unwell, I may not recognise the credibility of my decisions made when well, which could mean that a self-binding statement is needed, particularly if the medical team will adhere to specific information. On the other hand, it is difficult to predict all eventualities, and a plan made when well may not reflect the circumstances in the event of a crisis, and may be out of date or no longer wholly appropriate. Some flexibility may be beneficial - for me, my family and the medical team.	Y
2	The advance care plan was formulated when the person was well and thinking rationally.	Y
2	If it is written in a well state then it shows that the person has considered the best option for their mental health.	Y
2	When you are in either manic or depressive episode you are more likely to stop people helping and just want to push away any help.With depressive episode,sometimes you feel suicidal and want to be left alone to carry this out.With a manic episode you feel so fantastic that it's hard to believe anything is wrong at the time.	Y
2	Because if I were ill I would not be thinking straight	Y
2	Because not always lucid when unwell	Y
2	I can understand that a self binding statement is useful If I am really manic or deeply depressed. But there are levels when I can function mentally when mildly "ill"	Y

2	I have put somewhat instead of definitely because of concerns about the process of generating this document / directive in the first place. If it is a statutory 'tick box' exercise like the CPA sometimes is, there could be a rush to finalise it without the implications being fully thought through. It would need to be revisited regularly. An out of date document in this situation could be very difficult to work with for the service user and staff. There needs to be some process of considering all advance decisions to ensure that potential ethical or practical issues that may arise are addressed and thought through.	Y
2	It is possible that when unwell some aspect of care might emerge that would be beneficial but which contradicts the care plan. There needs therefore to be some flexibility.	Y
2	Statement is made when well. When I become ill I don't agree with my statement /care plan, even though the plan is in my best interests & will assist in my recovery. I chose somewhat rather than definitely in the event that rational wishes may have changed between the time of writing & becoming ill. They must be updated regularly.	Y
2	Maybe a decision i make is made when I'm not in s good place	Y
2	Because I have better insight into my condition when I am well.	Y
2	You have given permission to those contacts to positively help you.	Y
2	Being unwell makes you vulnerable so your previous choice would be in your own best interest I think your advance care plan should be updated regularly as treatment options can change	Y
2	Because when you are unwell you cant make the right decisions so you have to go with what you agreed in the first place	Y
2	I would have put 'definitely yes' but it may be that the plan was not up to date and not taking into account any more recent medication changes or recent personal circumstances. (For example, if the person had split with a partner they may not now want that person to be responsible for their finances and property).	Y
2	Generally yes, because in the throes of an episode I'm unlikely to accept I'm unwell. However, there are occasions when I have genuinely not been unwell, but my care-co has disagreed. In those instances, it is important that I am able to say 'I'm not following my plan' because I have good reason, and it should be respected.	Y
2	A tricky subject - lack of insight when ill is overruled by decisions made when well, but inevitably there will be complex cases.	Y
2	Advance plans are drawn up during stable episodes. Overruling during a manic or depressive negates the whole notion of advance planning	Y
2	Advanced planning is done so that when a person is unwell, others can know what the person wanted when well. Unfortunately the difficulty comes then, when deciding whether the person has improved and now has mental capacity to make new decisions.	Y
2	Any such plan needs to be made in a sound state of mind	Y
2	Because I am incapable of making rational decisions when I am unwell	Y
2	Because I believe judgement can be impaired when experiencing a bipolar episode and that the wishes of the person when they are healthy should be given greater weight than the wishes of the person when they are ill. However, it is sometimes not until the person is imminently faced with an intervention that the consequences become "real" enough for them to evaluate how they feel about it - it might be harder to imagine what you feel about a specific treatment option in advance (when healthy) than it is when that treatment is about to be imposed. Also, it might be difficult to assess whether somebody is fully "well" when they write their advance care plan eg. perhaps the person is a little hypomanic or somewhat depressed - not fully unwell but not fully well either - how is "wellness" defined?	Y
2	Because I believe that whether manic or depressed, I still have the mental capacity to make my own decisions.	Y
2	Because I can lie easily and convincingly when I am manic	Y
2	Because I know that when I am unwell, especially when I was manic in the past, I would become argumentative, not listen to the advice of family or friends.	Y
2	Because I wouldn't be in a position to make informed judgement or decisions.	Y
2	Because if I am unwell I will not see it clearly	Y
2	Because if they are unwell then it may be that their illness is clouding their judgement in a psychotic or manic episode say, but it it also possible that the person is ill, but also has capacity and has just changed their mind.	Y
2	Because in a manic episode, for example, the person can't make a good decision for themselves anymore. They can think they are OK.	Y
2	Because in an ill situation one might not be able to make the best decisions for oneself.	Y

2	Because it’s difficult to tell if I am being genuine	Y
2	Because not capable of making rational decisions when I'll.	Y
2	Because on a manic episode I get delusional and make bad decisions although this is tricky and a gray area.	Y
2	Because perception can change when unwell and thought processes can become muddled and confused	Y
2	Because the decisions were made when they were in a good frame of mind.	Y
2	Because unless circumstances had changed, then my mind must have. Meaning im not thinking straight. This is open to change, abuse and situation which can not be pre thought.	Y
2	Because when get unwell in the past have become delusional.	Y
2	Because when having a manic episode my daughter and husband recognise the symptoms before I do	Y
2	Because when I've been manic I haven't been my normal self and less rational than usual.	Y
2	Because when you are 'unwell' you are incapable of thinking in a coherent way.	Y
2	Because you presumably were in a rational "good space" when you wrote it. It needs to be periodically reviewed to make sure it meets your expectations.	Y
2	Being unwell can be cause for someone to disagree with current plan. However ensuring plan is reviewed on an appropriate time scale is vital. As the illness can change quickly. I argued when most ill as felt nothing would help me however i am grateful that i had amazing gp who helped to keep me safe	Y
2	Decisions when unwell may be poor.	Y
2	Depending on the state of mind if the person	Y
2	Depends how up to date the statement is, opinions change.	Y
2	Difficult to explain but whatever emotional state I'm at I feel like I always gave felt that way and it's hard to remember feeling another way. Sometimes keys to getting out of that state are only in that state and aren't visible at other times.	Y
2	During an unwell episode a patient is unable to experience clarity so it is a good idea to have this in writing.	Y
2	Enables good decisions to be made when feeling well	Y
2	Hopefully, you would have made the statement at a time when you were in good health and sound mind. When unwell, you can make irrrational decisions.	Y
2	I am a what if person. I can definitely imagine being against a lot of things if unwell/unrational, but whilst I am well, it is great to think rationally i.e. make the plan.	Y
2	I believe that the person affected should have some input into their treatment - not just dragged off to a psychiatrist to be stuffed up with pills, which ultimately do no good at all. (Sorry, this is probably a bit over the top but I'm really depressed at the moment - nothing appears to be helping.)	Y
2	I don't always think clearly when I'm in a very low mood or hypomanic episode	Y
2	I don't like the idea that it's 100% unchangeable but I can see the purpose	Y
2	I feel I would have more clarity and perspective on the situation when well compared to when I am unwell.	Y
2	I feel that the point of an Advanced Care Plan is to acknowledge that someone's capacity to make decision is reduced, and to override any potentially unsafe or unhelpful decisions one might make when unwell. Stating clearly that the Advance Care Plan is to be followed regardless of how one might feel when unwell seems a) necessary to avoid delayed decisions during illness, and b) important to convey how vital the contents of the document are when making decisions around future care. Knowing that I will be bound to the contents when incapacitated is highlighted by including a self-binding statement, and will force me to think extremely carefully about what I do and don't want.	Y
2	I have problems making logical choices when in extreme ends of spectrum.	Y
2	I know myself very well indeed. The well me knows what the unwell me needs. Any such provisions in might make in such an instance would have been so carefully considered before recording they would be my absolute wishes	Y
2	I loose touch with reality when I become unwell. My Advance Directive was constructed and agreed when I was well.	Y
2	I may be in a state where I cannot make rational decisions	Y
2	I think it's important as in times of psychosis or extreme mania it would be helpful	Y

2	I think that it should be respected as when I'm unwell I make unwise decisions. Although you should take into account how long ago it was made as there is a possibility that I could have changed my mind by then regardless of being unwell	Y
2	Ideas and thoughts are often very different when one is well and able to rationalise an appropriate plan which may then not be the case when they are ill.	Y
2	If a person put a 'self-binding statement' when they were in a non aggitated state in their advance care plan, then the 'self-binding statement' would hold weight when the individual was going through an episode. Again, in order for the self-binding statement to be upheld, it would have to be in agreement to what the medical profession would deam as necessary...as the medical professional can overrule individual's wishes in their care plan.	Y
2	If I am manic/mixed episode my ideas could be outlandish	Y
2	If I am unwell mentally could make wrong decisions under stress	Y
2	If I have pre arranged something when I am well , I can rest assured that if I do become unwell I will be looked after	Y
2	If its made when I am stable.	Y
2	If I've not got capacity, then the advance care plan ought to help me, that's why its put in place, whilst I'm well?	Y
2	If someone is able to change their mind on the care plan when they are unwell, then what is the point in it in the first place? It should state the patient's wishes for their care when they are well and in a stable state of mind. However there should be some sort of flexibility on this - possibly down to medical professional judgement	Y
2	If stated not to be hosptalizd then that is my wish and what I want, to be treated at home. Support from Cpn cat team and family , even if they say I sho Kid be in hospital	Y
2	If the decisions were made when I was of sound mind they should be listened to if not compleatly adhered to through my choice	Y
2	If the plan is made when the person is well and able to make rational decisions it should be respected.	Y
2	If you make a decision about treatment when you are well. Then change your mind when you are unwell. It should be ensured that you are not in a spiral of self destruct	Y
2	I'm not sure. I would hate it if I wrote something that was enforced which I then later disagreed with, but I would probably be disagreeing with it because I'm unwell! If the advance care plan is reviewed regularly, so that the patient can choose to make changes and remembers that it is there, then it is probably a good idea. But making someone adhere to the content of their care plan which they may have forgotten about a year or more later is not fair and could result in a lot of difficulties. For instance, if one of the people to be informed is a boyfriend / girlfriend and then the couple has since broken up, etc. Or agreeing to take a medication and then later on not being happy with the side effects. Then you wouldn't know if the patient was disagreeing because they are unwell or because they really shouldn't have these things in the plan.	Y
2	In episodes of mania you have no understanding of what is right for you	Y
2	In manic or depressive state beliefs and opinions about my own health and behaviour alter erratically and my ability to hold reasonable relationships with people and the world can be lost. That said, flexibility needs to be maintained. Time, space, condition and environment all play a part.	Y
2	In my experience, my thinking becomes skewed so I would disagree with certain boundaries that I would normally want to be in place. This would become difficult because of interpretation but even more so if the plan is out of date.	Y
2	It depends on how often a plan is reviewed by the patient & the professional - I think a review should be done every 2-3 years at the very least	Y
2	It has to be regular updated to make sure the persons wishes has not changed	Y
2	It is impossible for someone to anticipate all the possible circumstances that may arise, so the plan should not be absolutely binding. On the other hand there clearly arise circumstances under which the patient's best interests are to go with the plan.	Y
2	it reflects your thinking when of sound mind	Y
2	It should be binding otherwise it is useless	Y
2	It would be useful were I to be unable to make rational decisions due to illness eg if manic and thinking no treatment was needed, or depressed and suicidal	Y

2	It's made when your well but when your ill and your judgement is ill affected it's frustrating that you feel your not having a say in your care and wishes even though you are incapable of making good judgements at that time	Y
2	Its difficult to make rational and safe decisions when ill and changing the plan when in crisis, i believe, isn't well thought out and could lead to further difficulties	Y
2	My judgement would be too badly affected while I'm ill.	Y
2	not able to make a sensible decision.	Y
2	Only if it was written during a stable phase.	Y
2	Probably depends on personal circumstances. State of mind. Human rights!	
2	Sometimes it is not possible to make a rational choice.	
2	Sometimes when unwell become out of touch with reality and don't know what is best to do	
2	Sometimes when you are unwell you are unable to make rational decisions or think for yourself.	
2	the plan reflects hope when well and a aspiration when unwell.	
2	The plan should have been considered with support when the patient was at their most stable and informed. If an unwell person were to see a signed copy of their plan, it may help. In a deluded state paranoia can easily cloud everything. I firmly believe that deep down a person knows what is best for them and should be allowed to voice this and it should be respected by professionals as well as family members and friends.	Y
2	The treatment should always bear in mind the wishes of the patient when well and remember they are how a patient sees himself coming through an episode.	Y
2	the very fact that we are unwell can lead to making poor decisions and not being rational about what is needed to handle the situation until we are well enough to take care of ourselves and our own affairs again	Y
2	The whole point of doing a plan is for the forward planning when you are unable or unwilling to make those decisions.	Y
2	There must be some possibility for flexibility, or it could become distressing. It could even put people off writing the plan in the first place. Perhaps changes should be agreed with support from either family/GP/psychiatrist.	Y
2	They are unwell they don't necessarily know what is best for them	Y
2	This would be provided the care plan is up-to-date	Y
2	To help with care when unwell	Y
2	When a person is well they are in a good place to make decisions about their care and advance care plan. From personal experience when unwell this judgement is lost and you may not even be aware of what you are saying/doing.	Y
2	When I am unwell I am not necessarily in the right mind to make decisions that are for the best of my health and welfare and also that of my dependents. I will be concerned however, that if there was something I was strongly against and circumstances had changed, I would need someone like my carer to be able to back me up on my decisions.	Y
2	When I am unwell sometimes my judgement is very impaired so decisions I made when well would be more likely to be best.	Y
2	When I become unwell I tend to make very bad decisions and act irrationally. This would prevent that possibility arising.	Y
2	When ill I am not capable of clear logical thinking	Y
2	When unwell (especially if psychotic) is relatively easy to be frightened of particular treatment options and feel that they are being used not in your best interests. Paranoia can take over. Perhaps showing the unwell person their self binding statement could reassure them that they are being treated according to their wishes and help lessen feelings of persecution etc...	Y
2	When unwell views may change but probably not rationally.	Y
2	When unwell we don't 'think straight'. This would allow our well self to make the decisions.	Y
2	When unwell, decision making abilities can be distorted or not clear, and it may lead to bad decisions. Having a self-binding statement may prevent this from happening. However, if the person decides at times of crisis, that another plan might be more suitable, this self-binding statement may not work in their favour.	Y
2	When you are unwell you cannot think rationally	Y

2	When you are unwell you may have specific irrational beliefs about certain people. I often think certain people are against me but in reality are not.	Y
2	When you are unwell you often are not aware of what is ‘good’ for you. The decision you made when you are well should therefore be upheld. This would be very distressing though both to you, family and friends.	Y
2	When you become unwell you can lack insight into what treatment will make you well.	Y
2	When you're manic, you don't see things as they really are. You don't always know you're ill, or how ill you are. You can get paranoid, you might be really scared, or the opposite and think you're in heaven or something like that. Plus, the idea of going to a psychiatric hospital is terrifying to anybody. As is lack of control. As far as medication is concerned, I've no problem with a doctor being able to control what I take when I'm really ill, as they've always brought me back in the past.	Y
2	You may think you knpw best but it could be the mania or depression making you feel differently	Y
2	Your opinions are a lot different when unwell.	Y
2	You're not necessarily thinking in your normal mind when you're ill, so your judgement is impaired.	Y
2	Difficult to implement when at start of severe mania as I would not be in a state to follow instructions from familt/friends or medical team.	Y
2	It is important to have your independence promoted where possible. I would hate to feel that my opinions on my own care were not being listened to because of an episode	Y
2	This is a tricky area because potentially circumstances may have changed since the advance care plan was made and in some circumstances it could be a little out of date. E.g. Who is involved in care- someone could be experiencing relationship difficulties and not want the involvement of that person in their care plan, may have had a poor reaction to certain meds but not updated their plan to reflect that.	Y
2	Things change when you're unwell so what was in the plan might not be 100% applicable.	Y
2	This is a very difficult question that gets to the heart of the matter. When you are I’ll, in or nearing hospital, having a manic episode, then you are not in your right mind and not making sense. However you are still you and some things you can still deal with. It’s different each time. And hopefully it continues to be a learning curve where you gradually get better at managing your condition, so who is to say what the next time will be like, how I’ll you will be, what the medical staff will be like, what new drugs they might offer etc.. so giving up control in advance is a difficult decision to take.	Y
2	People's views and opinions change over time, so I feel that an Advance Care plan should include a review or renewal date. I would hate to think that my feelings may be ignored due to a document I signed years earlier or when my circumstances eg family, marriage etc were different.	Y
2	It may be difficult to foresee all circumstances exactly within pkan	Y
2	When you in any episode it will be difficult to agree / stick to an advanced care plan as your health feeling & thoughts will be very different to what was previously agreed	Y
2	If you're unwell, you don't feel the same way about yourself, treatment or recovery but that doesn't mean your new feelings are invalid. They should be considered but it should be remembered that you are unwell - especially if depressed as you are less likely to want to try for recovery in depressive episodes.	Y
3	Sanity can be a delusion so you my make plans which at the time seem good but in the heat of 'battle' are inapropriate	A
3	Last year I had ECT, another patient in the room next to me had it against her will under mental capacity act. We went for treatment together it was so distressing to see her have to go to through it. It first I thought it was horrible But it did help her recover and get discharged	A
3	I think it should be considered but a plan that was made say a year before may not be not be applicable	A
3	It depends. I may think going to a hospital is a good idea when I am well, but if I do end up going there when manic I may want to get out if treated in an unprofessional and unkind manner (i.e. no doctor telling them why I am there, attaching me to my bed, not allowing me to wear leggings under my hospital gown which makes me cold, not telling me when I'm allowed to shower etc.) Then I would prefer to go home with my spouse or go to a different type of facility where they can better look after patientes WITH bipolar (especially in manic cases in my case)	A
3	It's a tricky issue since there is no clear time when I would be stable enough to be truly objective. I think I'm a bit down at the moment. But am I? how does this affect my decision?	A
3	CHANGE OF MIND MUST BE ACKNOWLEDGED	A

3	This is difficult to comment on and I would be relying on those closest to me to interpret what would be closest to my best interests in this case.	A
3	Things can change	A
3	It depends how ill you are. Docs sometimes have to make decisions in difficult circumstances in order to help you. A self binding statement could get in the way. Then there is the question of legal responsibility and liability.	A
3	I'm not sure. I have been wrongly hospitalized when sedatives and my current antipsychotics could be tweaked so I'd stabilize at home. I'd want an advanced statement to refuse an admission if at all possible.	A
3	it depends how up to date the care plan is, so indicating the in the contents when it was written and so influencing the degree to which the statement should be followed or not. EG if admitted ro hospital a year after the statement date and things have changed for that peson then the statement will be 'out of date'. But if it's recently been written and as a binding statement there is more of a case for following it despite protestations against it. HOWEVER such decisions should ALWAYS be made in consultation with patient, CPN and/or psychiatrist (?) family/friends and GP. always respecting the patient	A
3	I think this varies from each individual.	A
3	It is hard to say. It depends how unwell you are in a particular circumstance. I have been unwell but still perfectly able to make my own decisions and on occasions I have been so unwell I've not been able to make my own decisions.	A
3	An elapse of time might render the plan not as effective as when drafted	A
3	i think doctors and family are better placed to judge in my case than i can be even when well as i have only had a serious episode once and cannot accurately predict what eventualities could occur if i became ill again. i would prefer to indicate my wishes in a written statement (rather than tick box form) primarily for my husband's reference and reassurance should i become ill, equipping him to liaise with doctors with out undue concern that he may be going against my wishes. if i had no partner or close family it would be more important to have a clearer plan that mental health teams could refer to. But either way this should not clutter or complicate their decision making during an emergency but might inform care once an emergency has passed / during prolonged treatment as well as giving them a clearer sense of tge person they are treating	A
3	Assuming the details within the plan have been regularly reviewed, updated and agreed to they should be upheld during a period of crisis if the individual is deemed to lack capacity. If the individual does have capacity and whilst in crisis does not agree to arrangements made within the plan, a conversation should take place between the individual and their consultant. It may be that a different course of treatment or different arrangements could be agreed upon. It would be important to establish the underlying reasons behind the individual disagreeing with their original plan. However, it would also need to be held in mind that during stages of relapse, particularly mania or hypomania, the individual may be unaware or in denial of their current mental health situation. At which point the plan agreed should be upheld in order to best support that individual. It is not a clear cut answer, but instead, stages, protocols and procedures should be put in place to clearly establish whether the individual has capacity to change their mind. It should also always be held in mind that before their illness, the individual is a person.	A
3	Being unwell doesn't mean I completely lose the ability to make decisions about everything - just that I need someone to check them for balance. It may be that reality doesn't match up to my imagination of how the care plan would work out so I would like to be able to change my mind, if I was able to justify my decisions and that justification makes sense to others.	A
3	Could have changed between making and becoming so unwell, but not recorded...due to practicalities of life, and thus may not be true wishes	A
3	Depends on the circumstances at the time eg Is treating doctor good, is staff respecting patient, is environment good or not, does family think the treatment is not right...	A
3	I might still have capacity to make a decision e.g. About which hospital to be admitted to, so the advance plan would only apply if I couldn't make a decision	A
3	When you are ill sometimes you think you are making rational decisions but you are not.	A
3	I think the service user should be respected whatever their thinking & behaviour is at the time. The proffesionals should respect the service user & the plan they originally wrote. If the plan includes things that can't be achieved anymore then that makes sense & can't be avoided. However if the plan was written when the service user was somewhat stable & able to understand then the service user should go along with the original plan despite their thinking & feelings towards it.	A

3	I would personally wanted to be treated fairly if I were unwell or well.	A
3	If it was a current "working document" approach then perhaps yes. If not, No as mood fluctuations affect decision making process.	A
3	It can be hard to distinguish whether or not a person's unwellness means that their wishes are unreasonable, I could be extremely manic but have a strong and legitimate reason for objecting that I've come to since making the advance decision. However I also see the reason in really securing safety.	A
3	Not sure how I will feel if I'm force to do or think a certain way when I'm I'll.	A
3	On the one hand, the person could lose touch with reality when they're unwell and not feel the same way as they normally do when they're well. On the other hand, a person could sign something like this and then, while still in their right mind, change their mind about treatment but fall ill before they could change the document, causing them to be subjected to treatment they don't want.	A
3	How do you know they were of fit mind when they wrote it? How do you know someone else has not befriended that person for financial gain?	A
3	People do change their mind a lot that is separate from being unwell.	A
3	The difficulty around this is knowing yourself is hard enough at times let alone trusting someone else to judge if your unwell enough to action any plans.	A
3	Things change and sometimes it's difficult to guess what might happen in a hypothetical situation	A
3	Things may change	A
3	Were I manic I might still have insight, were I psychotic probably not.	A
3	When you become unwell with either a high or a low episode your way of thinking changes.	A
3	Would be dependant on the circumstance.	A
3	You are allowed to change your mind	A
3	You are entitled to change your mind.	A
3	I think it would be hard to agree a definition of 'when they are unwell'. Other people may think I am unwell and I might disagree.	A
3	Decisions have to be made at the time.	A
3	I have doubts as even if in depression/ mania I would have my own opinions	A
3	It’s so hard with Bipolar and my family are very controlling. I am afraid what could happen and knowing I were committed to it forever could be daunting	A
3	They may disagree with it when well but have overlooked need to update it. Somewhat uncomfortable with this idea if not the notion of self binding statements generally.	A
3	I might rationally change my mind and wouldn’t want to be restricted by an outdated plan.	A
3	I don’t think i would agree with many of my decisions while having an extreme episode	A
3	Because people's ideas when they are high do not reflect how they are normally	A
3	I think it depends entirely on the individual knowing how he/ she behaves during acute episodes. In some cases it might be helpful.	A
4	Circumstances in hospital can be different from expected depending on staff	N
4	I just think it's risky when we start stopping people from the right to withdraw consent	N
4	I think this allows for the individual, who is after all the expert of their own condition, to change their mind. This allows for involvement which is essential and gives the user/ client / patient ownership and self-respect in their care, recovery and eventual integration back into their life and home.	N
4	I don’t think anyone should be forced to undertake medical care. Sectioning is obviously necessary when one can no longer care for oneself but I am horrified by the notion that I would be treated in a way that was against my will at the time.	N
4	It is likely that following or during any episode I may wish to change some approaches, particularly if what I thought would work does not	N

4	I am wary of being hospitalised when I am manic, because I do not feel that this is a destructive part of my illness: in fact I enjoy it, am happy, productive and move my life on in important ways, ways which I can't do in hospital. However, I am well aware that psychiatric professionals all see mania as very destructive, even though my most destructive behaviour is by far when I am severely depressed. So I assume that a psychiatrist would probably attempt to get me to agree to hospitalisation/ treatment for manic episodes as well as depressive, which I do not want, but could not convince a psychiatrist of.	N
4	Not having been in this situation personally, it is very difficult to tell. This is an extremely emotive issue, especially where a Section is involved. It relates essentially to respecting the individual as well as their prior wishes, and, of course, there is the element of lacking insight when unwell - that may have been there during Advance care planning. My first instinct would be to follow the wishes of the person in the present day, however I can see how an Advanced care plan might be thought to be appropriate to follow, given need for medication despite lack of insight. This can be a real dilemma. Advance Care Plans can be outdated, (can also be put together more by the influence and wishes of care staff than the individual,) and can fly in the face of what the service user is stating in the present moment can be tremendously disempowering, potentially damaging trust and involving essentially not listening to the patient concerned. This, I find a bit worrying- however I am also aware that without medication, (e.g. the one agreed to in Advance Care Planning) the person concerned could become even more unwell. Should Advance Care Planning be used in this way, it is of utmost importance that the service user concerned be made fully aware of the 'self-binding' nature.	N
4	A person may change his or her mind, but not have changed the plan. They need to be asked.	N
4	People still have valid views even when manic. What people really need most of all is an excellent psychiatrist or care coordinator who provides continuity over time and sites of care and for mh services to be far better resources and humane than they are. People will then trust them in a crisis rather than using an inevitably limited piece of paper. Also it is not so much myself I want to bind as to bind the health professionals in hospital to providing good humane well resources and insightful care	N
4	Psychosis can result in rash and loss of reality therefore patient may not be of sound mind - from my experience excepting treatment from pyschitrist has been the best way forward for me and a quicker recovery. However, I would not be happy with ECT	N
4	Doesn't sound right	N
4	Because circumstances change and I want to b able to change my mind rather than tied down to inflexible decision which I may not agree wit at the time.	N
4	Because someone should not be bound by what they have said previously, even if they are currently unwell and not making characteristic decisions. It is a matter of civil liberties.	N
4	It is impossible to say whether being unwell makes you alter your decision when you shouldn't or being unwell gives you a different perspective on what you want or need	N
4	I feel an individuals wishes should be respected although there is a point when an individual becomes too unwell and can not make decisions for themselves	N
4	I think how a person is feeling in a crisis is valid. As it's valid at the time, the advanced statement might be temporarily invalid.	N
4	A plan that is written when I am well is a good idea BUT I may have changed my mind about things and not updated the original plan. I feel that if a doctor could measure my response, talk to relatives etc. and override my original plan if I choose this should happen.	N
4	Because unless it's updated regularly it may be wrong and out of date	N
4	Because we're all entitled to change our minds, that shouldnt change because we're ill.	N
4	Being unwell does not necessarily mean that you are unable to make a decision that is not right for you. Considering what is best for someone is subjective and it would be against one's human rights to not allow someone to change their mind about what is happening in a specific situation when a plan is made on what to do in a hypothetical one.	N
4	Being unwell doesn't always mean you are not able to revise your ideas of what you would want from such a plan at the time.	N
4	Even when I'm mad, I'm still a human and have the right to make decisions even if they are bad ones.	N
4	Factors can change.	N
4	I can usually reason quite well even when in depression and if the plan seemed reasonable once it will still be reasonable.	N

4	I have never been in that situation so I wouldn't want it.	N
4	I might have changed my mind at some aspect	N
4	I think that even when ill I would like to be able to change my mind.	N
4	I wouldn't want to be forced against my will	N
4	If you are unwell you should still have the right to discuss your care and the reasons you made requests in your advice care plan	N
4	It would be better that all involved in the original to agree on what to do.	N
4	Moods can change opinions	N
4	Not every mental health episode is the same	N
4	Situations change, people evolve and develop. Care plans should be reviewed often	N
4	So complicated. I have a big thing about consent and if you are overriding consent in the moment I feel pretty uncomfortable but difficult as when manic, so hard to know real feelings	N
4	The advance care plan should be regularly reviewed	N
4	Trust is the issue. Sadly what rules the NHS is economics so however well intentioned staff are they are likely to have to confirm to what the latest cuts dictate.	N
4	when one is unwell - to a degree (in my experience) judgement is impaired - however the statement needs to be flexible enough in wording so that I as an individual feel respected even when unwell and no one is talking over me or down to me - but is reasoning with me. Even when I went high on one occasion I was listening to my therapist who reasoned with me. If I refuse medication - that I had agreed to in my statement - only in that case would I wish to be ruled over but still with respect. no need for superior approach	N
4	You may change your mind when actually faced with being ill in a way you hadn't imagined	N
5	Because I am an intelligent person and have developed a reasonably good amount of insight over my 60 years ..... and would not wish to give anyone else carte blanche over my own autonomy	N
5	Situations and relationships change and the capacity to make decisions can be assessed on a case by case basis. Independent people are vital as friends and family have too many pre conceived ideas and possible vested interests often emotions can prevent fair treatment for the ill person for instance embarrassed relatives preferring hospital admissions or sections to impose strong drugs to make patient more compliant or obedient to family wishes. Family may love the ill person but wish to run their life rather than allow the person to make their own decisions which family consider invalid or flawed rather than different from the family norm.	N
5	There may be a long time between preparing Advance Car Plan and it being needed	N
5	When I have been ill before I still have been lucid in my mind but I have needed people to realize my speech has stopped because of fear but I could express my wishes by writing it down if someone had encouraged me to do so. I think it would be the same situation.	N
5	This is an extremely dangerous area and goes back to what I was saying about how vulnerable bipolar sufferer are. I have experience of the NHS and being unwell. Just because a sufferer is unwell does not equate to lacking capacity to make decisions. Perhaps some of their judgement is impaired but they still know what foods they prefer, what colours they like. It's a very difficult and vulnerable area. Any self binding statement I think is dangerous and should be avoided at all costs. Rather statements of wishes/feelings. There is the distinct possibility a sufferer makes a self binding statement and then changes their mind, has mental capacity and is then overruled. It goes back to the MH Act, but the people in charge of making the decisions about capacity are not also best placed or indeed right in their decisions. Nothing should be self binding.	N
5	Not empowering or respectful	N
5	Even if you are unwell you may have changed your mind and not updated your plan. Each person's circumstances would be different	N
5	I am against any such plan. As I mentioned every episode has been different and handled in a different way. I do recognise that some people need more help than others. I have good insight into my condition.	N
5	A persons best wishes should be respected in any state. It's hard to say really. I'm not sure that it will come to this right now.	N
5	At the point when writing the advance care plan, the person may not have enough information about what their options might be during a future episode.	N

5	Because a person's needs can change. I believe that a person should not be treated against their will, and they have the right to decide differently even when others judge them not able to make a decision. I think you are taking advantage of a person's vulnerability when in an episode of bipolar and as we know it does change. Bipolar is about changing moods. The intelligence of the person is unimpared if you take the time to communicate through the fogging of the mood state. If a person without bipolar was upset and temporarily incoherent you would not judge them to be without capacity. You would know to give them space and time to calm down. Having the threat of your own personal autonomy removed is worse than being depressed, that is not something that resolves by itself. And being at the mercy of a less than perfect system where you are judged on a bad day means people will wait until things get really bad because the terror of being judged incapable is worse for them.	N
5	Because even ill people can change their mind rationally.	N
5	Because even when severely ill a person can think for themselves and has a right to do so. It should act as a guide be owned by the person it is for	N
5	Because you feel out of control as it is. Sometimes different things work and if you're bound to something that *may* work when you're incapable of making the decisions for yourself then fine to use it. Otherwise allow somebody to feel as though they have some sort of control over their own destiny and give them the sense that they can overcome this	N
5	Circumstances and perspectives change	N
5	I trust clinicians to be bound by their duty to treat me according to the immediate and presenting symptoms, not some guess made the previous year. They medicate, care, and where possible seek insight from friends and family. Sad fact, bipolar can smash up the world and leave one picking up the pieces at a later time. So too can a broken back or shattered knees. Help me face the daunting haul back to autonomy following an episode, not shackle and fetter me with red tape.	N
5	I would like the freedom to make my own decisions with the help of my family and don't like the idea of being tied to any previous decisions.	N
5	If a person says they wanted to stay home to be treated but are clearly a danger to themselves or others hospital and care is the best place.	N
5	The self-binding statement may have been advised at a particular time, when that person was to ill to make an informed decision.	N
5	Things change and the patients wishes should always be listened to and respected.	N
5	Unless I am sectioned or under arrest nobody has any legal right to compel me to do anything regardless of what I said yesterday.	N
